# Estradiol inhibits HIV-1_BaL_ infection and induces CFL1 expression in peripheral blood mononuclear cells and endocervical mucosa

**DOI:** 10.1038/s41598-022-10163-6

**Published:** 2022-04-13

**Authors:** N. Verma, S. Mukhopadhyay, P. Barnable, M. G. Plagianos, N. Teleshova

**Affiliations:** Center for Biomedical Research, Population Council, 1230 York Ave., New York, NY 10065 USA

**Keywords:** HIV infections, Cell signalling, Cytoskeleton, Immunology, Molecular biology

## Abstract

An inhibitory effect of estradiol (E2) on HIV-1 infection was suggested by several reports. We previously identified increased gene expression of actin-binding protein cofilin 1 (CFL1) in endocervix in the E2-dominated proliferative phase of the menstrual cycle. Actin cytoskeleton has an integral role in establishing and spreading HIV-1 infection. Herein, we studied in vitro effects of E2 on HIV-1 infection and on CFL1 expression to gain insight into the mechanism of HIV-1 inhibition by E2. E2 dose-dependently inhibited HIV-1_BaL_ infection in peripheral blood mononuclear cells (PBMCs) and endocervix. In PBMCs and endocervix, E2 increased protein expression of total CFL1 and phosphorylated CFL1 (pCFL1) and pCFL1/CFL1 ratios. LIMKi3, a LIM kinase 1 and 2 inhibitor, abrogated the phenotype and restored infection in both PBMCs and endocervix; inhibited E2-induced expression of total CFL1, pCFL1; and decreased pCFL1/CFL1 ratios. Knockdown of CFL1 in PBMCs also abrogated the phenotype and partially restored infection. Additional analysis of soluble mediators revealed decreased concentrations of pro-inflammatory chemokines CXCL10 and CCL5 in infected tissues incubated with E2. Our results suggest a link between E2-mediated anti-HIV-1 activity and expression of CFL1 in PBMCs and endocervical mucosa. The data support exploration of cytoskeletal signaling pathway targets for the development of prevention strategies against HIV-1.

## Introduction

Our recent study identified an inverse association between serum estradiol (E2), the most potent estrogen, and ex vivo HIV-1 infection in endocervix and ectocervix^1^. Analysis of endocervical transcriptome from subjects in the same cohort identified 202 differentially expressed genes (DEGs) during proliferative vs. secretory phase of the menstrual cycle^2^. Among the top DEGs, mRNA expression of actin-binding protein cofilin 1 (CFL1; Log2FC = 0.9) was upregulated in the E2-dominated proliferative phase of the cycle.

Cofilin 1 (CFL1) is a 21 kDa actin-binding protein involved in regulation of in-bound and out-bound HIV-1 and cell-associated HIV-1 transfer^3–7^. Early binding of HIV-1 to CD4 and CXCR4/CCR5 coreceptors initiates actin polymerization that correlates with phosphorylation of CFL1 (pCFL1; inactivated state) and promotes HIV-1 entry^8^. Dephosphorylation of CFL1 (activated state) promotes intracellular viral migration through CFL1-mediated actin treadmilling. CFL1 phosphorylation is regulated by signaling kinases including LIMK/ERK^7,9^. In cycling cells, CFL1 is constitutively dephosphorylated^10^. In resting CD4^+^ T cells, which are resistant to HIV-1 infection, CFL1 is largely phosphorylated hindering viral post-entry migration^6,10^. In productively infected 293T cells and primary T cells, Nef-mediated phosphorylation of cofilin is observed^11,12^. Interestingly, lower pCFL1 levels were detected in resting CD4^+^ T cells derived from HIV-1-infected subjects than in healthy subjects^6,13^. Also, an association between low pCFL1 level, high viral load and low CD4 counts in HIV-1 infected subjects was reported^13^. Based on these findings, the level of pCFL1 was suggested to be a candidate biomarker of disease progression^6,13^.

This study was designed to explore the role of CFL1 in E2-mediated effects on HIV-1 infection in PBMCs and endocervical mucosa. We hypothesized that E2 would inhibit in vitro HIV-1 infection, induce CFL1 gene expression, and increase CFL1 total protein and pCFL1 expression.

## Materials and methods

### Reagents

HIV-1_BaL_ was generated in CD8-depleted PBMCs in the presence of 10 nM retinoic acid (Sigma-Aldrich, St Louis, MO), 20 U/ml IL2 (NIH) and 50 ng/ml of anti-CD3 monoclonal antibodies (clone OKT3; e-Bioscience, San Diego, CA) and titered in TZMbl cells as previously described^14^. Lamivudine (3TC) (Sigma-Aldrich) was used as a control for inhibiting HIV-1_BaL_ infection. PBMCs and endocervical tissues were incubated with E2 (Sigma Aldrich). The following inhibitors or activators of E2 signaling were used: selective estrogen receptor modulator (SERM) Raloxifene (Sigma-Aldrich), and LIMKi3 (LIMK1/2 inhibitor blocking CFL1 phosphorylation; Tocris, Minneapolis, MN).

Antibodies (Abs) used in Western Blot were rabbit monoclonal anti-human CFL1 (D3F9), pCFL1 (77G2), β-Actin (D6A8); AKT (pan) (C67E7) and pAKT (D9E) (Cell Signaling Technology, Danvers, MA); HPRT1 (Sigma-Aldrich); and HRP-conjugated goat anti-rabbit IgG (secondary Ab) (Abcam, Cambridge, MA).

Antibodies used for Immunofluorescence staining (IF) were rabbit monoclonal anti-human CFL1 (EP6376) (Abcam); mouse monoclonal HIV-1-p24 (24-4)-AlexaFluor 488 (AF488) (Santa Cruz Biotechnology); mouse monoclonal anti-human CD3 (OKT3)-AF488, IgG-AF488 (eBM2a), goat anti-rabbit secondary Ab AF568, IgG isotype controls (Invitrogen, Carlsbad, CA) and DAPI (Sigma-Aldrich). FVS 780-APC-Cy7 (FVS780) (Live/Dead dye) was used for flow cytometry.

### Effect of E2 and E2 signaling modulators Raloxifene and LIMKi3 on HIV-1_BaL_ infection in PBMCs

PBMCs were isolated from buffy coats of anonymous healthy HIV-1 uninfected human blood donors (New York Blood Center, New York, NY) using a Ficoll-Paque Plus (Cytiva, Marlborough, MA) density gradient. PBMCs were cultured in cRPMI [RPMI 1640 (Corning, Corning, NY) with 10% charcoal stripped FBS (Atlanta Biologicals, Flowery Branch, GA), 2 mM l-Glutamine (Gibco, Thermo Fisher Scientific, Waltham, MA), penicillin (100 U/ml) and streptomycin (100 µg/ml) (Corning)]. PBMCs (2 × 10^6^/ml in duplicate wells; 48-well plate) were incubated with E2 (100–10,000 pg/ml) for 48 hours (h) and then challenged with HIV-1_BaL_ (1000 TCID_50_/10^6^ cells) for 4 h. PBMCs were washed with DPBS (Cellgro, Mediatech, Manassas, VA) and cultured in cRPMI for 14 days. The supernatants were collected on days 0, 3, 7, 11 and 14 for HIV-1 *gag* quantitative reverse transcription PCR (qRT-PCR). 3TC (10 µM) was used as control. In selected experiments, PBMCs were incubated for 3 h with Raloxifene (1 µM) or LIMKi3 (1, 5 and 10 µM) followed by 48 h incubation with or without E2 10,000 pg/ml, then challenged with HIV-1_BaL_ and cultured for 14 days as described above. Raloxifene, LIMKi3 and 3TC and E2 were added to the cultures on days 3, 7 and 11.

### Effect of E2 and E2 signaling modulator LIMKi3 on HIV-1_BaL_ infection in endocervix

Human endocervical tissues without gross pathological changes were obtained from routine hysterectomies through the National Disease Research Interchange (NDRI, Philadelphia, PA) and transported overnight in RPMI 1640 (Corning). Tissues were derived from subjects 32–50 years old (13 Caucasian; 4 Black; 1 Hispanic and 1 unknown ethnicity). Polarized endocervical explant cultures (duplicates) were set up in cDMEM [DMEM containing 10% Charcoal stripped FBS, 2 mM l-Glutamine and penicillin (100 U/ml) and streptomycin (100 µg/ml)] as previously described^15,16^. E2 (100–10,000 pg/ml) was added to the bottom chamber of transwells. Following 48 h incubation, the explants were challenged with 500 TCID_50_ HIV-1_BaL_ (10 µl total volume) for 4 h followed by DPBS washes and cultured for 14 days. To determine the effect of E2 signaling modulators on infection, explants were treated with LIMKi3 (10 µM) for 3 h followed by 48 h incubation with or without E2 10,000 pg/ml, challenged with HIV-1_BaL_ and cultured for 14 days. The supernatants were collected on days 0, 3, 7, 11 and 14 for HIV-1 *gag* qRT-PCR. 3TC (10 µM) was used as control in selected experiments. E2, LIMKi3 and 3TC were added to the cultures on days 3, 7 and 11.

### Effect of CFL1 knockdown on HIV-1_BaL_ infection in PBMCs

PBMCs were plated in 24-well plate (10^6^ cells/ml) in cRPMI 24 h prior to transfection. The cells were transfected with CFL1 small interfering RNA (siRNA) (Thermo Fisher), Trilencer-27 Universal scrambled negative control siRNA duplex (Origene, Rockville, MA) and Trilencer-27 HPRT Positive control siRNA duplex (Origene), using Viromer Green transfection reagent (Origene) following the manufacturer’s protocol for suspension culture cells. Media was changed after overnight incubation. 48 h post-transfection, PBMCs were either incubated with E2 10,000 pg/ml for additional 48 h or left untreated. 96 h post-transfection, the cells were challenged with HIV-1_BaL_ 1000 TCID_50_/10^6^ cells and cultured for 14 days. The supernatants were collected on days 0, 3, 7, 11 and 14 for HIV-1 *gag* qRT-PCR. E2 was added to the cultures on days 3, 7 and 11.

### HIV-1 *gag* qRT-PCR

The infection was monitored in the supernatants using HIV-1 *gag* quantitative reverse transcription PCR (qRT-PCR) (lower limit of quantification [LLOQ] 2000 copies/ml; values below LLOQ were set to 2000^1/√2^ = 215.86), using SYBR FAST One-Step qRT-PCR (Kapa Biosystems, Wilmington, MA). The primers used were forward primer 5′ GGTGCGAGAGCGTCAGTATTAAG 3′ and reverse primer 5′ AGCTCCCTGCTTGCCCATA 3′. The cycling conditions were 1 cycle at 42 °C for 5 min, 1 cycle at 95 °C for 5 min, and 40 cycles at 95 °C for 3 s and 60 °C for 20 s. HIV-1 *gag* qRT-PCR was performed using ViiA 7 real-time PCR system (Applied Biosystems, Carlsbad, CA) and data were analyzed using ViiA 7 software (Applied Biosystems). Dissociation curves were generated to verify the absence of nonspecific amplification. Results were analyzed with the standard curve method using pNL(AD8) 10^7^–10 copies/μl (11346; NIH AIDS Research and Reference Reagent Program, Germantown, MD)^14^. SOFT and CUM endpoint analyses of infection level based on HIV-1 *gag* copies on days 3–14 of the culture were performed as previously described^14^. SOFT is an estimation of HIV-1 *gag* copies at the start of the stationary phase of virus growth and CUM is the cumulative number of HIV-1 *gag* copies^14,17^.

### RNA isolation and gene expression qPCR

RNA isolation from PBMCs and explants were done according to the manufacturer’s protocol using Qiagen RNeasy mini and RNeasy Fibrous Tissue kit (Qiagen, Hilden, Germany), respectively. Primers for CFL1 were purchased from Bio-Rad (Hercules, CA). 200 ng of RNA was used to synthesize cDNA using High-capacity RNA-to-cDNA kit (Applied Biosystems). Gene expression was monitored by quantitative real time-PCR (qPCR) using SYBR FAST qPCR kit (Kapa Biosystems) and ViiA 7 real-time PCR system.

### Immunofluorescence staining (IF)

Following culture, PBMCs were plated on poly-l-Lysine coated slides (Polysciences Inc. Warrington, PA) for 1 h, washed with DPBS (Corning), fixed (15 min) with 4% paraformaldehyde (PFA), permeabilized using permeabilization buffer (Thermo Fisher Scientific) and blocked with 2% BSA. This was followed by incubation with primary CFL1 (0.108 µg/µl) and HIV-1-p24 -AF488 (0.2 µg/µl) Abs in 2% BSA overnight at 4 °C and then by incubation with secondary Ab in 2% BSA and DAPI (1X) for 90 min. After DPBS washes and air-drying, cells were mounted in Prolong mounting medium (Invitrogen) and sealed with coverslips. The images were acquired on Nikon Eclipse Ti2 at 20X magnification and analyzed using NIS Elements v5.31.01. Total fluorescence intensity of CFL1 and p24 staining (3 fields per condition) was normalized relative to fluorescence intensity of IgG control.

Following culture, explants were washed in DPBS and placed in the Histosette biopsy cassettes (Thermo Fisher Scientific), fixed overnight at 4 °C in 4% PFA followed by 10 min wash under running water. The cassettes were placed in 70% ethanol before paraffin embedding and sectioning at Molecular Cytology Core, MSKCC, New York. Slides were heated at 60 °C for 30 min followed by × 3 washes in Histo-Clear II (National Diagnostics, Atlanta, GA) for 10 min each, × 2 washes in absolute alcohol for 10 min each, 5 min washes each in 95%, 70% and 50% ethanol. The slides were rinsed in deionized water and rehydrated in DPBS for 10 min. Then slides were placed in pre-heated (92–95 °C) antigen retrieval buffer (Millipore, Burlington, MA) for 5 min and cooled down for 5 min at room temperature followed by water and DPBS rinses. Next, sections were permeabilized for 10 min, blocked with 5% BSA for 30 min and incubated with primary CFL1 Ab (0.108 µg/µl), and CD3-AF488 (0.2 µg/µl) Ab in 2% BSA overnight followed by three 10 min DPBS washes, and secondary Ab and DAPI (1X) staining for 90 min. After three 10 min DPBS washes, sections were air-dried and mounted in Prolong mounting medium. The images were recorded on Nikon Eclipse 90i at 10X magnification and analyzed using ImageJ (NIH). Total fluorescence intensity of CFL1 and p24 staining (3 fields per section) was normalized relative to fluorescence intensity of IgG control.

### CFL1 and pCFL1 Western Blot (WB) and densitometry

#### PBMCs

PBMCs used for WB were cultured as described above. PBMCs were incubated with E2 for 48 h or pre-treated with LIMKi3 for 3 h followed by 48 h incubation with E2. The cells were then either collected for WB analysis or challenged with 1000 TCID_50_ HIV-1_BaL_. PBMCs challenged with HIV-1_BaL_ were cultured for 7 or 14 days and collected for WB analysis.

In siRNA experiments, transfected PBMCs were collected 48 h post-transfection for WB analysis or incubated with or without E2 for additional 48 h. The cells were then collected for WB or challenged with 1000 TCID_50_ HIV-1_BaL._ PBMCs challenged with HIV-1_BaL_ were cultured for 3 days and collected for WB analysis.

PBMCs were gently washed with ice cold DPBS, lysed using RIPA lysis and extraction buffer (Thermo Fisher Scientific) supplemented with protease inhibitor cocktail (Sigma-Aldrich) according to manufacturer’s instructions. Whole cell extracts (WCEs) were stored at − 20 °C. Protein concentrations were measured by Bradford method (Bio-Rad). 60–120 µg of protein was mixed with NuPAGE LDS sample buffer (Thermo Fisher Scientific) and β-Mercaptoethanol (Thermo Fisher Scientific) and heated for 10–15 min at 95 °C. WCEs were resolved on standard NuPAGE 4–12% Bis–Tris SDS-PAGE in MOPS SDS buffer (Thermo Fisher Scientific) and transferred to PVDF membrane using Transblot (Bio-Rad). Membranes were blocked with standard 5% non-fat dry milk (Sigma-Aldrich) dissolved in DPBST (0.1% Tween-20, Sigma-Aldrich). Blots were incubated with CFL1, pCFL1, HPRT1 and β-Actin Abs in DPBST overnight at 4 °C followed by HRP-conjugated secondary Ab incubation for 1 h at room temperature. The membranes were developed using the ECL system (Thermo Fisher Scientific) and analyzed using Amersham Imager 600 (Cytiva).

#### Endocervical tissue

Endocervical tissue explants were incubated with E2 (100 and 10,000 pg/ml) for 48 h. The explants were then either collected for WB analysis or challenged with 500 TCID_50_ HIV-1_BaL_. Explants challenged with HIV-1_BaL_ were cultured for 7 days and collected for WB analysis.

The explants were washed with ice cold DPBS, snap frozen in liquid nitrogen, cut, and lysed in RIPA lysis and extraction buffer supplemented with protease inhibitor cocktail to prepare WCEs. Bradford assay was used to measure protein concentration. 60–120 µg of protein was mixed with NuPAGE LDS sample buffer and β-Mercaptoethanol and heated at 95 °C for 10–15 min. WCEs were resolved on NuPAGE 8–16% Tris Glycine gel in Tris Glycine buffer (Thermo Fisher scientific). The gel/blot transfer and Ab probing were done as described above.

Protein bands were quantified and normalized by β-actin using densitometry method (Image Studio Lite v5.2 software (LI-COR Biosciences)).

### Flow cytometry

Uninfected and HIV-1_BaL_ infected PBMCs were incubated with E2, Raloxifene or LIMKi3 as described above. Untreated PBMCs and 16% PFA treated cells were included as controls. The cells were stained with FVS780, fixed with 4% PFA, and acquired on a BD LSR II (BD Biosciences) and analyzed by FLOWJO 8.8.6 software.

### MTT assay

Endocervical tissue viability post-treatments with LIMKi3 for 3 h and/or E2 for 48 h was tested using thiazolyl blue tetrazolium bromide (MTT) assay^18^. Untreated tissues and 16% PFA treated tissues were included as controls. Briefly, explants were washed and cultured in cDMEM in presence of 0.5 mg/ml MTT (Sigma-Aldrich) for 2 h at 37 °C. The explants were then incubated overnight in 1 ml of methanol in the dark. Readings from 200 µl of extracts (in triplicates) were recorded using Emax Spectrophotometer (Molecular Devices, San Jose, CA) at 570 nm (OD_570_) and viability was determined by normalizing the optical density of the formazan product by the dry weight of the explants.

### Luminex

Cytokines and chemokines (CC/CKs) in supernatants collected from endocervical tissue cultures after E2 treatment and 500 TCID_50_ HIV-1_BaL_ challenge were analyzed on days 3 and 7 using the Human Cytokine Magnetic 25-Plex Panel Kit (IL1RA, IL-1α, IL-2R, IL-2, IL-4, IL-5, IL-6, IL-7, IL-10, IL-12, IL-13, IL-15, IL-17, TNFα, CCL11, GM-CSF, IFNα, IFNγ, CCL2, CCL3, CCL4, CCL5, CXCL8, CXCL9, and CXCL10) (Thermo Fisher Scientific).

### Statistics

Log-normal generalized linear models were utilized for the analysis of HIV-1_BaL_ infection in PBMCs and endocervix. Kruskal–Wallis test with Dunn’s multiple comparisons was used for viability, gene expression, and PBMC WB densitometry data analysis.

Wilcoxon Matched Pairs Signed Rank t test was used for endocervix WB densitometry and fluorescence intensity data analysis in endocervix and PBMCs. Kruskal–Wallis test with Dunn’s multiple comparisons and Wilcoxon Matched Pairs Signed Rank t test were used for Luminex data analysis. The Wilcoxon Matched Pairs Signed Rank t test results were not adjusted for multiple comparison as these analyses are exploratory.

## Results

### E2 inhibits HIV-1_BaL_ infection in PBMCs and endocervix in a dose-dependent manner

**Figure 1:**
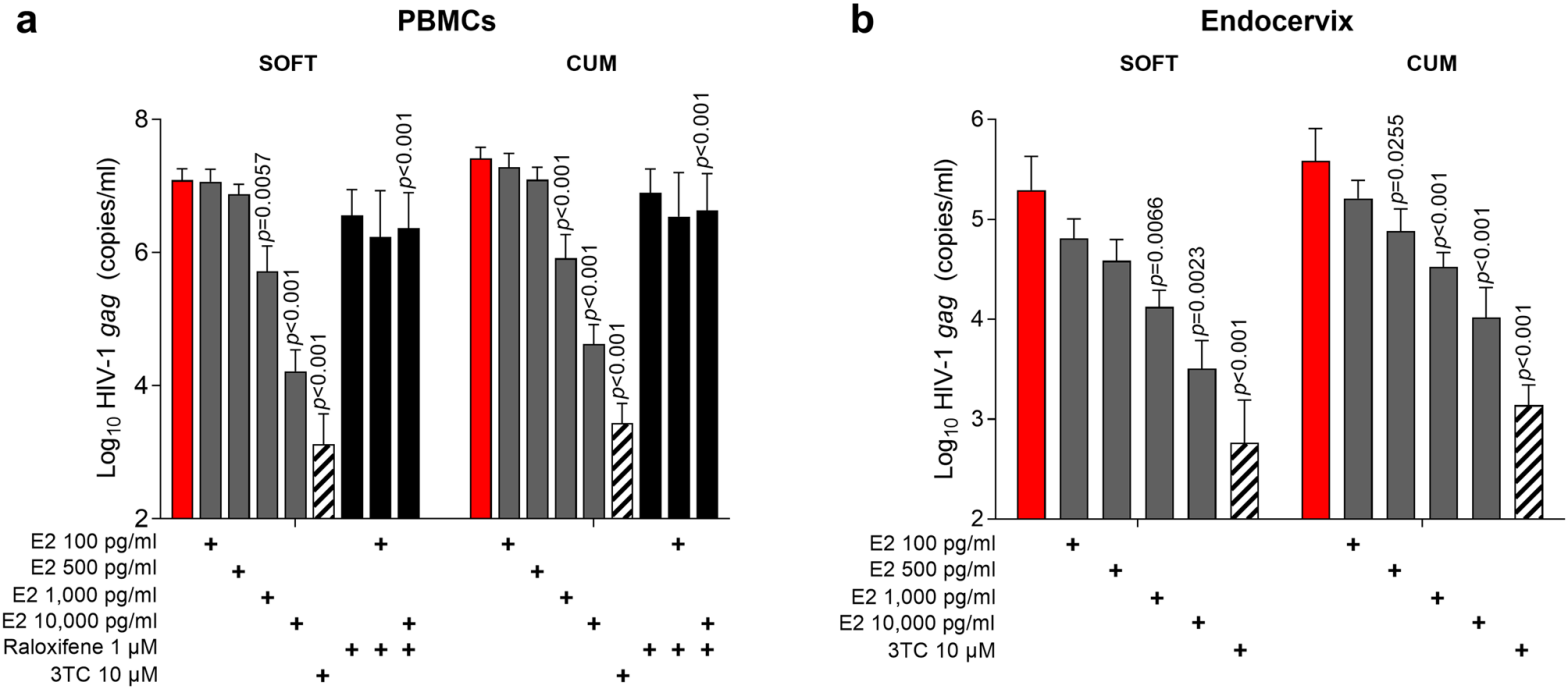
E2 inhibits HIV-1_BaL_ infection in PBMCs and endocervix in vitro. **(a)** PBMCs and **(b)** endocervical explants were incubated with E2 (vs. untreated control) for 48 h followed by 1000 TCID_50_/10^6^ cells or 500 TCID_50_/explant HIV-1_BaL_ challenge and then cultured for 14 days with repeated addition of E2 on days 3, 7 and 11. Raloxifene and 3TC were included in selected experiments. Infection was monitored by HIV-1 *gag* qRT-PCR. Shown are Log10-transformed SOFT and CUM analyses (Mean ± SEM, D3-14) of (**a**) 10 experiments (1 µM Raloxifene and 10 µM 3TC conditions were included in 4 experiments) and of (**b**) 9 experiments (10 µM 3TC was included in 2 experiments). The significant *p* values represent comparisons between E2 and untreated control; 3TC and untreated control; Raloxifene + E2 and E2.

To determine the effect of E2 on HIV-1_BaL_ infection in PBMCs and endocervical mucosa, PBMCs and endocervical explants were challenged with HIV-1_BaL_ and cultured in the presence of E2. In selected experiments, Raloxifene and 3TC control were included. E2 at non-toxic concentrations dose-dependently inhibited HIV-1_BaL_ infection in both PBMCs and endocervix (Fig. 1a,b; Supplementary Fig. S1 a,b). Viral growth kinetics in individual experiments are presented in Supplementary Fig. S2a,b. Expectedly, higher infection level was observed in PBMCs than in endocervix, likely due to higher challenge dose and higher target cell numbers. Consistent with published data^19^, Raloxifene, a SERM that blocks Estrogen receptor (ERα), reverted E2-mediated HIV-1_BaL_ inhibition at the non-toxic 1 µM concentration (Supplementary Fig. S1 a,b).

### Effect of E2 on CFL1 mRNA expression in PBMCs and endocervix

We analyzed effect of E2 on CFL1 mRNA expression in uninfected and HIV-1_BaL_ infected PBMCs and in uninfected endocervical tissues. CFL1 expression post-E2 treatment tended to be higher than in untreated controls (Supplementary Fig. S3). However, no significant changes in CFL1 expression were observed after 48 h of E2 treatment in uninfected PBMCs and endocervix (Supplementary Fig. S3a). An increase in CFL1 mRNA expression in HIV-1_BaL_ infected PBMCs post-E2 (1000 pg/ml) treatment was observed on day 3 of the culture. No changes in CFL1 gene expression in infected PBMCs were observed on day 7 of the culture (Supplementary Fig. S3b).

### E2 induced CFL1 protein expression coincident with decreased p24 expression

**Figure 2:**
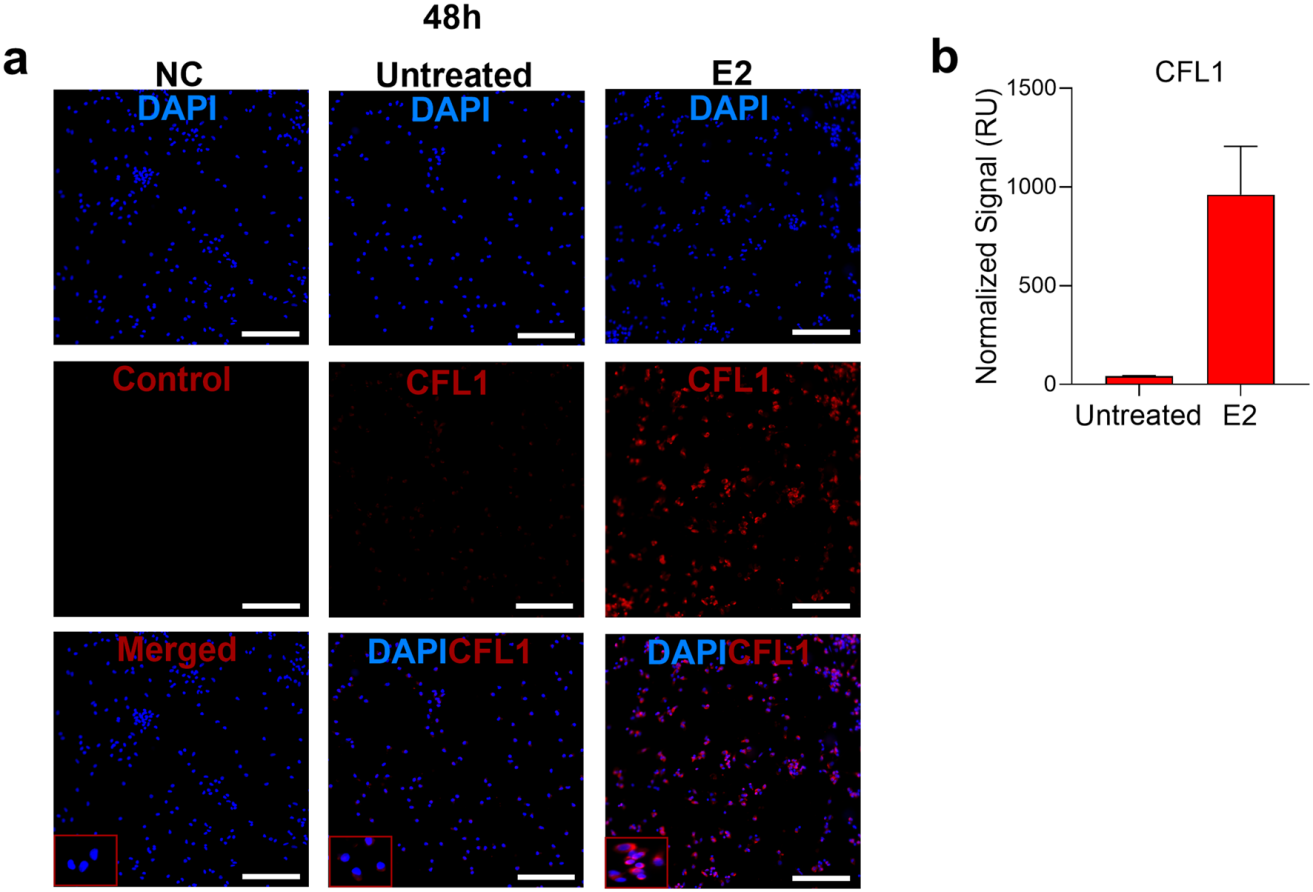
E2 induces CFL1 expression in uninfected PBMCs. **(a)** PBMCs were incubated with E2 10,000 pg/ml (vs. untreated control) for 48 h and then transferred to poly l-lysine coated slides for 1 h. PBMCs were then fixed, permeabilized and stained with rabbit anti-human CFL1 Ab followed by goat anti-rabbit AF568 Ab and DAPI. Rabbit IgG and goat anti-rabbit AF568 Ab were used as negative controls (NC) for CFL1 staining in untreated PBMCs. Single experiment representative of 3 experiments is shown (original magnification 20X; scale bar is 100 μm). **(b)** Shown is the normalized fluorescence intensity of CFL1 staining in relative units (RU) (Mean ± SEM; 3 experiments).

**Figure 3:**
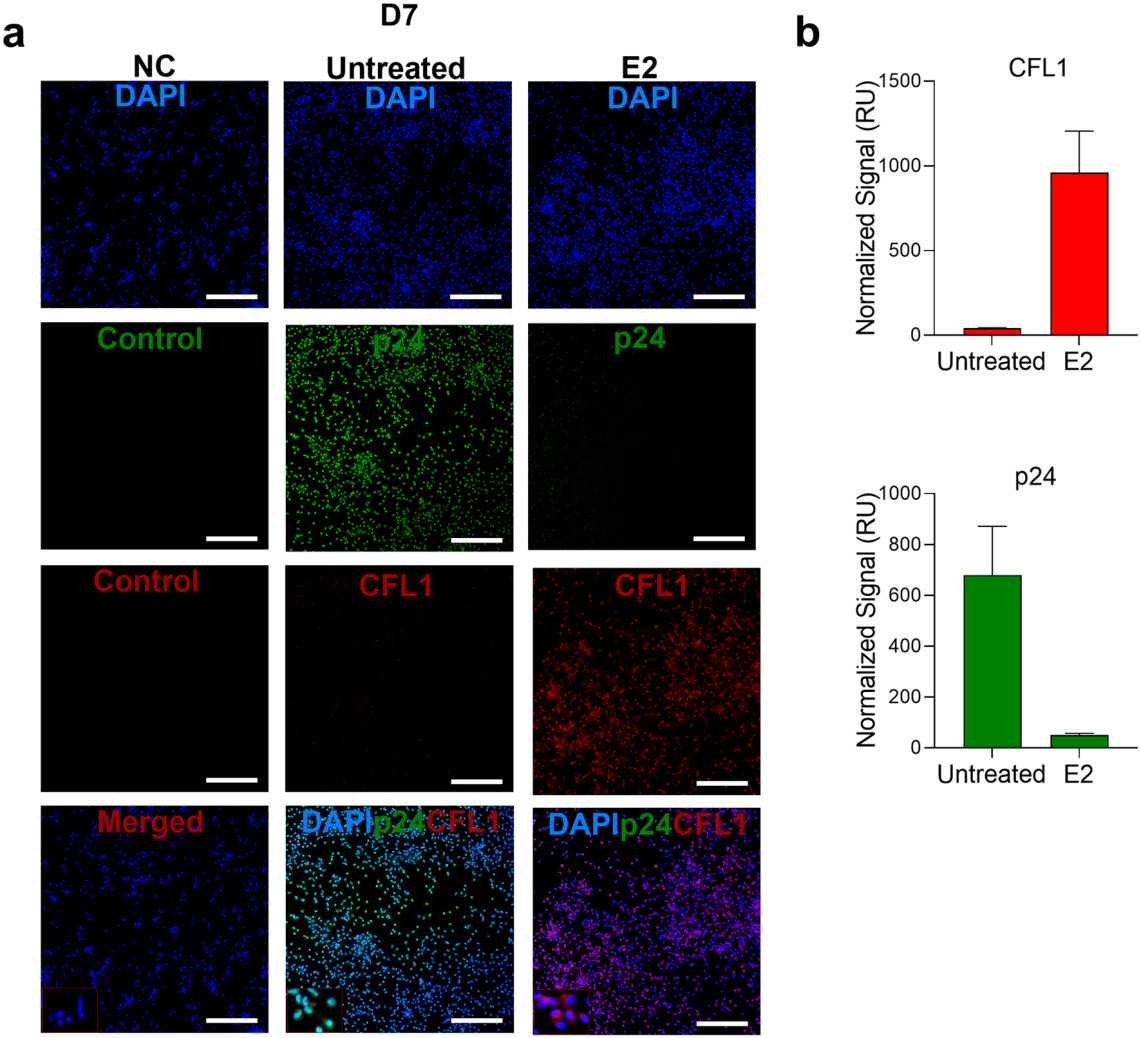
E2 induces CFL1 expression and inhibits p24 expression in HIV-1_BaL_ infected PBMCs. **(a)** PBMCs were incubated with E2 10,000 pg/ml (vs. untreated control) for 48 h followed by 1000 TCID_50_/10^6^ cells HIV-1_BaL_ challenge, cultured for 7 days and then incubated on poly l-lysine coated slides for 1 h. PBMCs were then fixed, permeabilized and stained with rabbit anti-human CFL1 Ab and mouse anti-HIV-1-p24 AF488 Ab followed by goat anti-rabbit AF568 Ab and DAPI. Rabbit IgG and goat anti-rabbit AF568 Ab were used as negative controls (NC) for CFL1 staining in uninfected untreated PBMCs. Uninfected untreated PMBCs were used as a NC for p24 staining. Single experiment representative of 3 experiments is shown (original magnification 20X; scale bar is 100 μm). **(b)** Shown is the normalized fluorescence intensity of CFL1 and p24 staining in relative units (RU) (Mean ± SEM; 3 experiments).

**Figure 4:**
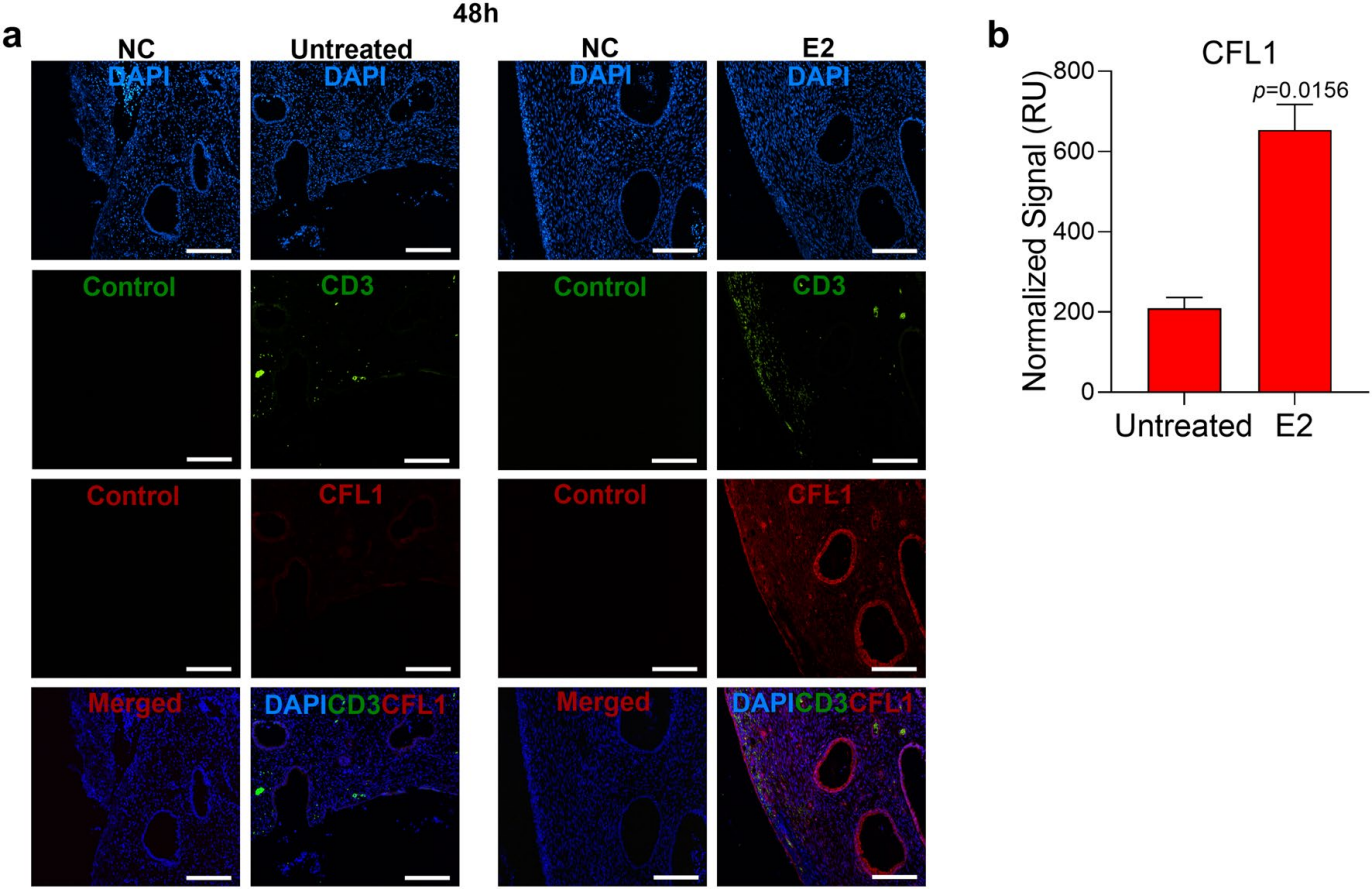
E2 induces CFL1 expression in uninfected endocervix. **(a)** Endocervical explants were incubated with E2 10,000 pg/ml (vs. untreated control) for 48 h and then paraffin embedded. The sections were stained with mouse anti-human CD3 AF488, rabbit anti-human CFL1 Ab followed by goat anti-rabbit AF568 Ab and DAPI. Rabbit IgG, goat anti-rabbit AF568 Ab and mouse IgG AF488 were used as negative controls (NC) for CFL1 and CD3 staining. Single experiment representative of 7 experiments is shown (original magnification 10X; scale bar is 100 μm). **(b)** Shown is the normalized fluorescence intensity of CFL1 staining in relative units (RU) (Mean ± SEM; 7 experiments).

**Figure 5:**
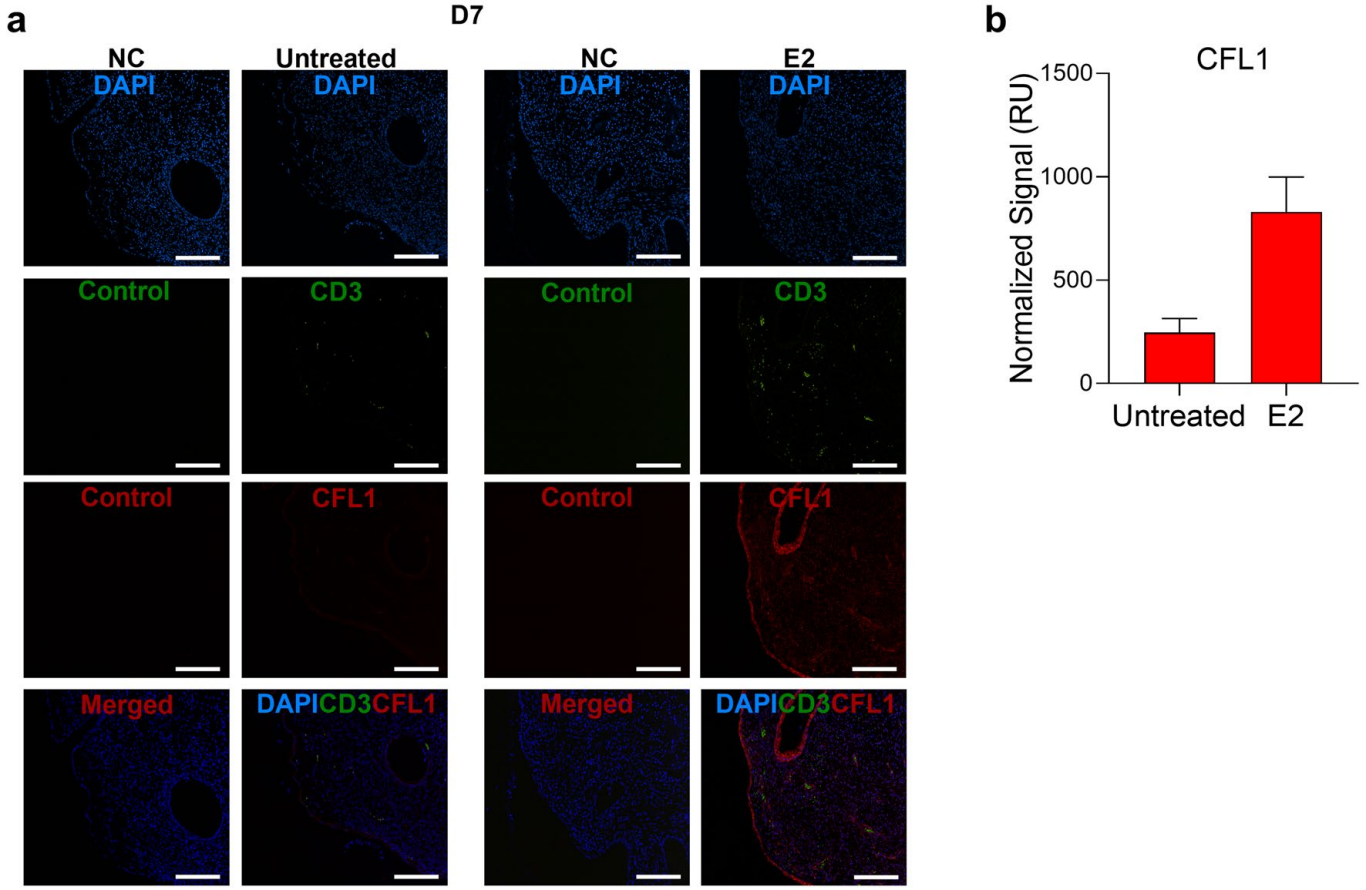
E2 induces CFL1 expression in HIV-1_BaL_ infected endocervix. **(a)** Endocervical explants were incubated with E2 10,000 pg/ml (vs. untreated control) for 48 h followed by 500 TCID_50_/explant HIV-1_BaL_ challenge. Tissues were cultured for 7 days and then paraffin embedded. The sections were stained with mouse anti-human CD3 AF488, rabbit anti-human CFL1 Ab followed by goat anti-rabbit AF568 Ab and DAPI. Rabbit IgG, goat anti-rabbit AF568 Ab and mouse IgG AF488 were used as negative controls (NC) for CFL1 and CD3 staining. Single experiment representative of 5 donors/experiments is shown (original magnification 10X; scale bar is 100 μm). **(b)** Shown is the normalized fluorescence intensity of CFL1 staining in relative units (RU) (Mean ± SEM; 5 experiments).

Next, we determined if exposure to E2 changes CFL1 expression at the protein level. Immunofluorescence staining demonstrated low baseline CFL1 expression in uninfected and HIV-1_BaL_ infected PBMCs. E2 treatment strongly induced CFL1 expression (Figs. 2, 3). Consistent with E2-mediated HIV-1_BaL_ infection inhibition detected by qRT-PCR, we observed decreased p24 expression in HIV-1_BaL_ infected and E2-treated PBMCs (Fig. 3). Similar to the results in PBMCs, low baseline CFL1 expression was detected in uninfected and HIV-1_BaL_ infected endocervical tissues along with robust induction post-E2 treatment (Figs. 4, 5). CFL1 was expressed ubiquitously, including CD3^+^ T cells (Figs. 4, 5).

A quantitative fluorescence intensity analysis also demonstrated E2-induced increase in CFL1 expression in PBMCs and endocervix (Figs. 2, 3, 4 and 5), which was statistically significant in endocervix (Fig. 4). The analysis also demonstrated a decrease in p24 staining intensity in E2-treated PBMCs (Fig. 3). This decrease was not statistically significant.

### E2 increases expression of total CFL1, pCFL1 and pCFL1/CFL1 ratios in PBMCs and endocervix

**Figure 6:**
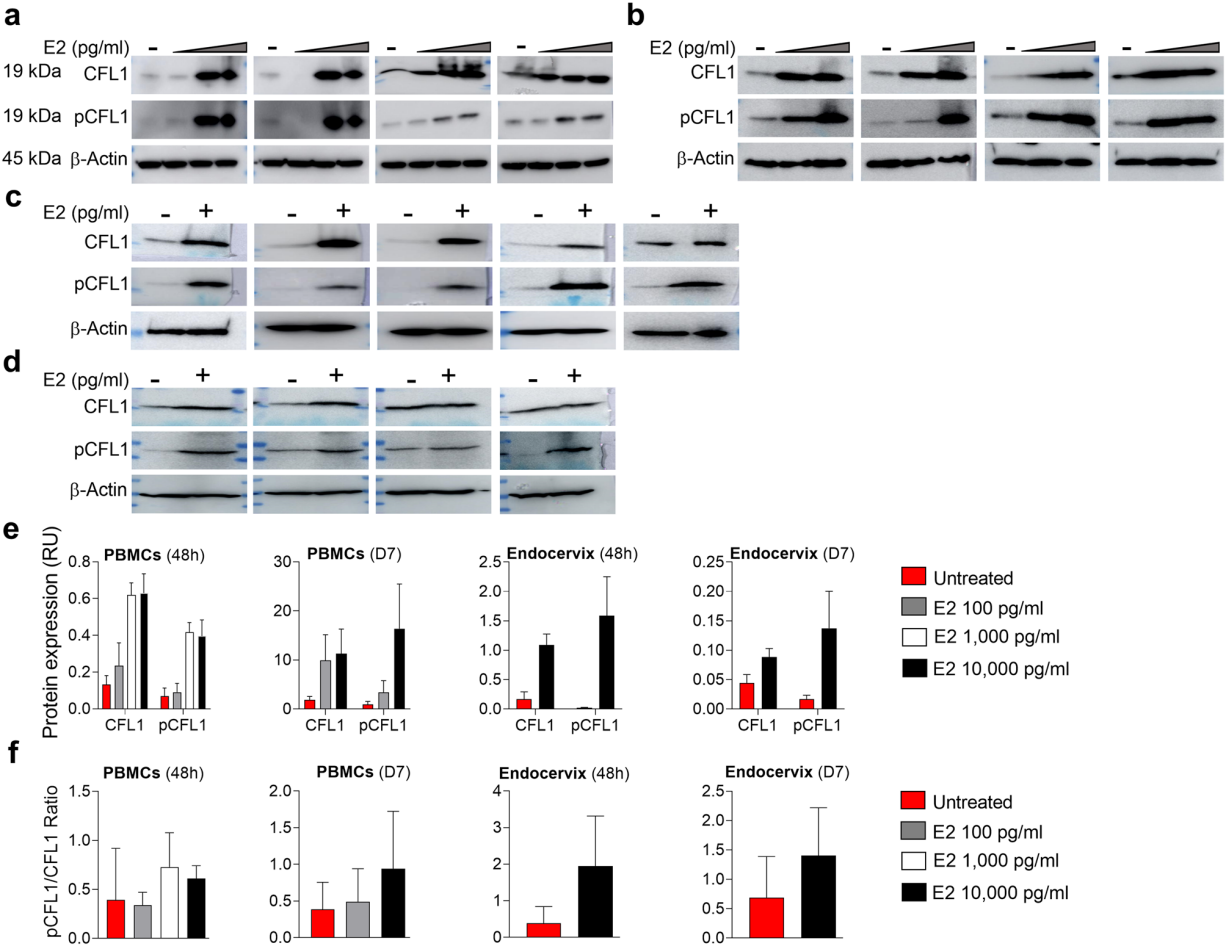
E2 induces total CFL1 and pCFL1 expression in PBMCs and endocervix and increases pCFL1/CFL1 ratio. PBMCs were **(a)** incubated with E2 100, 1000, 10,000 pg/ml (vs. untreated control) for 48 h (4 experiments) or **(b)** incubated with E2 100 or 10,000 pg/ml, challenged with 1000 TCID_50_/10^6^ cells HIV-1_BaL_ and cultured for 7 days (4 experiments). WCEs (60 µg) were probed for CFL1 and pCFL1. β-actin (60 µg) was used as internal control. **(c)** Endocervical explants were incubated with E2 10,000 pg/ml (vs. untreated control) for 48 h (5 experiments) or **(d)** incubated with E2 10,000 pg/ml (vs. untreated control), challenged with 500 TCID_50_ HIV-1_BaL_ and cultured for 7 days (4 experiments). WCEs (60–120 µg) were probed for CFL1 and pCFL1. β-actin (60–120 µg) was used as internal control. **(e)** Expression of CFL1 and pCFL1 in experiments presented in (**a**–**d**) was normalized relative to β-actin expression and plotted; and **(f)** pCFL1/CFL1 ratios were calculated and plotted. Shown are Mean ± SEM. For each experiment, CFL1, pCFL1 and β-actin blots were developed in parallel. Uncropped blots are presented in Supplementary Fig. S4a–d.

In agreement with IF results, WB analysis revealed an increase in CFL1 and pCFL1 expression in uninfected and HIV-1_BaL_ infected PBMCs and endocervix following E2 treatment, with more variation detected in infected endocervix (Fig. 6a–d). Quantification of bands by densitometry supported this observation and showed an increase in CFL1 and pCFL1 post-E2 treatment (Fig. 6e). The analysis also revealed higher pCFL1/CFL1 ratios following E2 treatment than in untreated controls (Fig. 6f; Supplementary Fig. S4a–d). However, the changes in total CFL1, pCFL1 and ratios were not statistically significant.

### LIMKi3 reverts E2-mediated anti-HIV-1_BaL_ activity in PBMCs and endocervix and decreases expression of total CFL1, pCFL1 and pCFL1/CFL1 ratios in PBMCs

**Figure 7:**
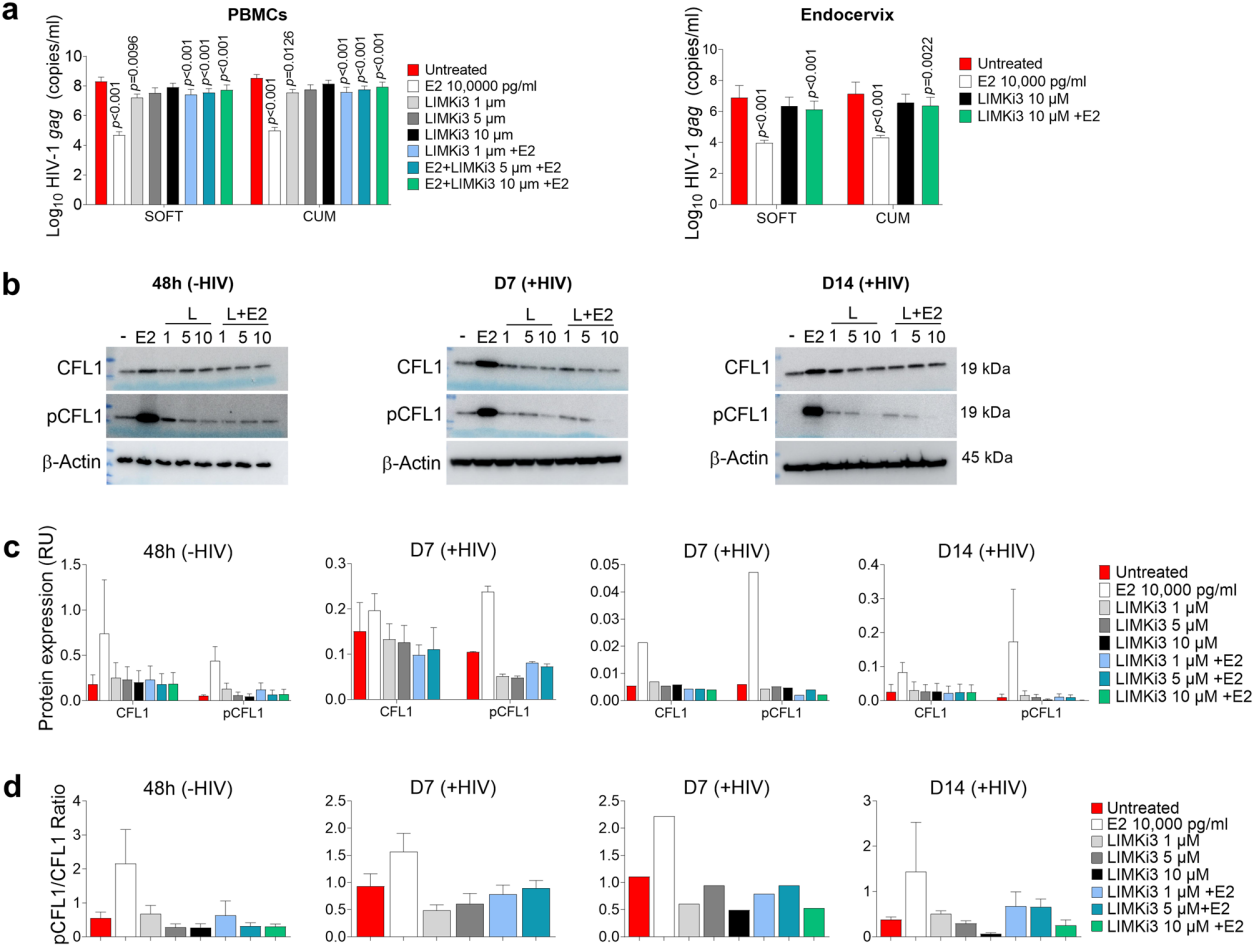
LIMKi3 reverts E2-mediated anti-HIV-1_BaL_ activity in PBMCs and endocervix and decreases expression of total CFL1, pCFL1 and pCFL1/CFL1 ratios in PBMCs. **(a)** PBMCs and endocervical explants were incubated with LIMKi3 for 3 h prior to 48 h incubation with E2 (vs. untreated control) followed by 1000 TCID50/10^6^ cells or 500 TCID_50_/explant HIV-1_BaL_ challenge and 14 days culture. E2 and LIMKi3 were added on days 3, 7 and 11 of culture. Infection was monitored by HIV-1 *gag* qRT-PCR. Shown are Log10-transformed SOFT and CUM analyses (Mean ± SEM, D3-14) (5 experiments in PBMCs; 4 experiments in explants). *p* values represent comparisons between E2 vs. untreated control, LIMKi3 vs untreated control and LIMKi3 + E2 vs. E2 condition. **(b)** PBMCs were incubated with 1–10 μM LIMKi3 and E2 10,000 pg/ml followed by HIV-1_BaL_ challenge (as in **a**). WB analysis was done before challenge and on day 7 and 14 post HIV-1_BaL_ challenge. WCEs (60 µg) were prepared at indicated time points and probed for CFL1 and pCFL1. β-actin (60 µg) was used as internal control. A single representative experiment of 48 h set (4 experiments), D7 set (3 experiments; LIMKi3 at 10 μM was included in a single experiment) and D14 set (3 experiments) are shown. **(c)** Expression of CFL1 and pCFL1 in experiments presented in (**b**) was normalized relative to β-actin expression and plotted; and **(d)** pCFL1/CFL1 ratios were calculated and plotted. Shown are the Mean ± SEM. As only one experiment in D7 set included LIMKi at 10 μM, this experiment is shown as a separate graph. For each experiment, CFL1, pCFL1 and β-actin blots were developed in parallel. Uncropped blots are presented in Supplementary Fig. S6a–c.

To explore the role of pCFL1 in E2-mediated anti-HIV-1 activity, PBMCs and endocervical explants were challenged with HIV-1_BaL_ in the presence of E2, LIMKi3 (CFL1 phosphorylation inhibitor), or E2 and LIMKi3. No cytotoxicity at the tested LIMKi3 concentrations was detected (Supplementary Fig. S5). LIMKi3 reduced E2-mediated anti-HIV-1 activity in PBMCs and in endocervix (Fig. 7a) and decreased E2-induced phosphorylation of CFL1 at all analyzed time points in uninfected (48 h) and infected (D7 and D14) PBMCs (Fig. 7b; Supplementary Fig. S6a–c). LIMKi3 did not change expression of total CFL1 as compared to untreated control. However, a reduction of E2-induced total CFL1 expression in the presence of LIMKi3 was detected (Fig. 7b; Supplementary Fig. S6a–c). Densitometry analysis supported these observations and demonstrated decrease in CFL1, pCFL1 expression and in pCFL1/CFL1 ratios in LIMKi3 + E2 conditions as compared to E2 only conditions (Fig. 7c,d). These results were not statistically significant. The specificity of LIMKi3 was checked using AKT and pAKT Abs (Supplementary Fig. S7).

### Knockdown of CFL1 reduces total CFL1 and pCFL1 expression in PBMCs and reverts E2-mediated anti-HIV-1_BaL_ activity

**Figure 8:**
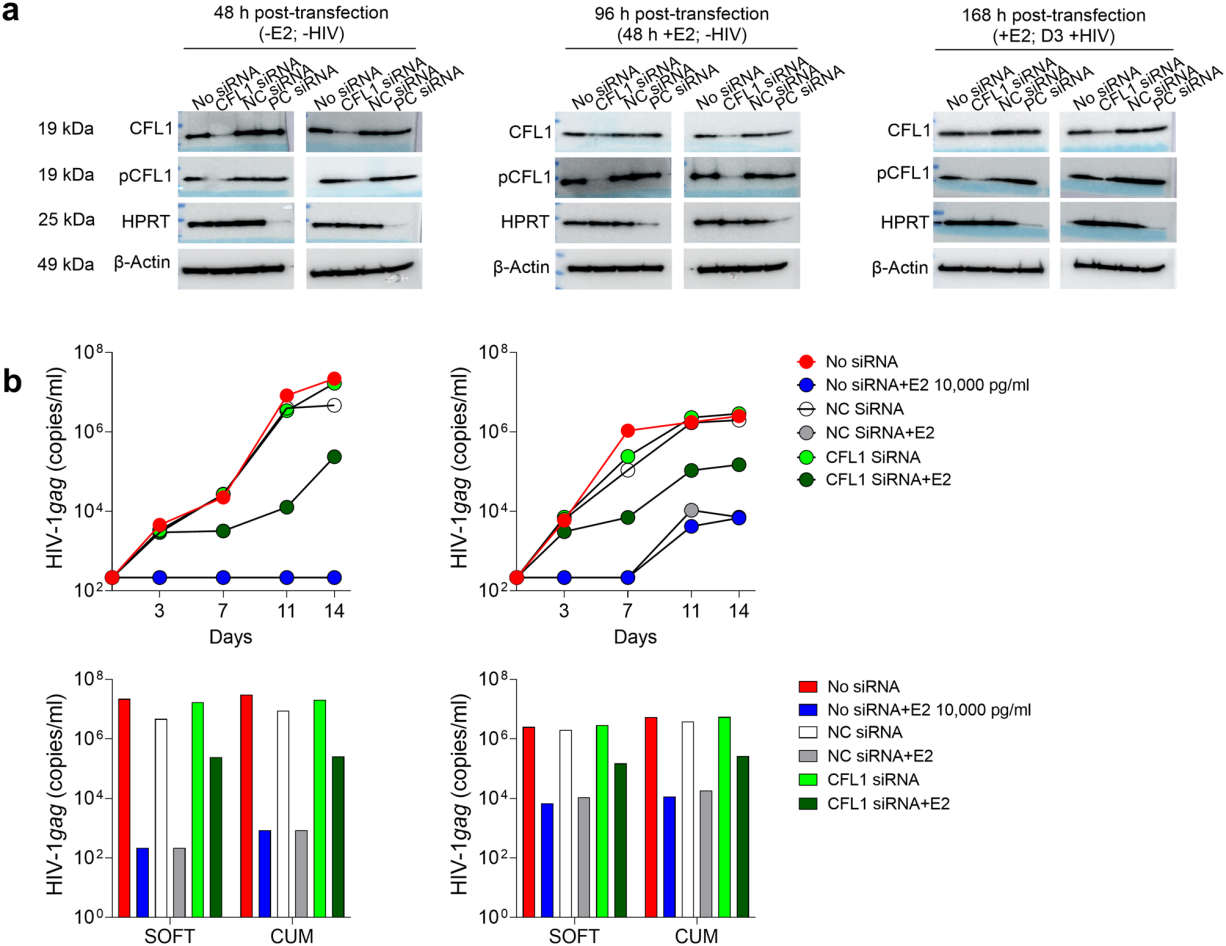
Knockdown of CFL1 reduces total CFL1 and pCFL1 expression in PBMCs and reverts E2-mediated anti-HIV-1_BaL_ activity in PBMCs. **(a)** PBMCs (10^6^ cells/ml) were transfected with CFL1 siRNA, Trilencer-27 Universal scrambled negative control siRNA duplex and Trilencer-27 HPRT Positive control siRNA duplex using Viromer Green transfection reagent. 48 h post-transfection, PBMCs were split in two groups and either incubated with E2 10,000 pg/ml for additional 48 h or left untreated. 96 h post-transfection, PBMCs were challenged with 1000 TCID_50_/10^6^ cells HIV-1_BaL_ and cultured for 3 days. WB analysis was done at 48 h, 96 h and 168 h post-transfection (2 experiments). WCEs (120 µg) were prepared at indicated time points and probed for CFL1 and pCFL1. β-actin (120 µg) was used as internal control. Uncropped blots are presented in Supplementary Fig. S8. (**b**) Transfected PBMCs were incubated with or without E2 10,000 pg/ml for 48 h followed by HIV-1_BaL_ challenge as described in (**a**) and 14 days culture. E2 was added on days 3, 7 and 11 of culture. Infection was monitored by HIV-1 *gag* qRT-PCR. Shown are the viral growth kinetics (HIV-1 *gag* copy numbers/ml) over 14 days of culture in individual experiments (Mean ± SEM of replicates) and Log10-transformed SOFT and CUM analyses (Mean ± SEM, D3-14) of 2 experiments. *NC siRNA* negative control/Trilencer-27 Universal scrambled negative control siRNA duplex, *PC siRNA* positive control/trilencer-27 HPRT Positive control siRNA duplex.

To confirm involvement of CFL1 in E2-mediated HIV-1 inhibition, we conducted CFL1 knockdown experiments. Transfection of PBMCs with CFL1 siRNA resulted in decreased total CFL1 and, expectedly, decreased pCFL1 protein level 48 h post-transfection as compared to control conditions (untransfected cells and cells transfected with control siRNAs) (Fig. 8a; Supplementary Fig. S8). PBMCs transfected with CFL1 siRNA were incubated with E2 for additional 48 h and then challenged with HIV-1_BaL_ and cultured for 14 days. We observed decreased CFL1 and pCFL1 protein expression at the time of HIV-1_BaL_ challenge (96 h post-transfection and 48 h post-E2 treatment) and on day 3 of culture after HIV-1_BaL_ challenge (168 h post-transfection) (Fig. 8a; Supplementary Fig. S8a–c).

PBMCs transfected with CFL1 siRNA, control siRNA and untransfected PBMCs demonstrated similar HIV-1_BaL_ infection level. Consistent with data presented in Fig. 1, E2 inhibited HIV-1_BaL_ infection in untransfected PBMCs and also in control siRNA transfected cells. CFL1 knockdown partially reverted this phenotype and resulted in diminished anti-HIV-1 activity of E2 (Fig. 8b).

### Effect of E2 on CC/CK concentrations in HIV-1_BaL_ infected endocervical tissue cultures

To further explore the effects of E2 in mucosa, we analyzed tissue CC/CK profile following E2 treatment. CC/CK concentrations in untreated and E2-treated HIV-1_BaL_ infected tissue supernatants collected on day 3 of the culture were similar. Expectedly, concentrations of several CC/CK (IL1RA, IL7, CXCL8, CXCL10 and GM-CSF) were higher in untreated condition on day 7 vs. day 3. CXCL10 and CCL5 concentrations in day 7 E2-treated culture supernatants were significantly lower than in untreated supernatants (Supplementary Fig. S9).

## Discussion

This study demonstrates a link between E2-mediated inhibition of HIV-1_BaL_ infection and increased expression of total CFL1, pCFL1 as well as increased pCFL1/CFL1 ratios in PBMCs and endocervix.

Our experimental approach focused on repeated addition of E2 to PBMCs and endocervix before viral challenge and during culture. This approach was rationalized to mimic continuous exposure to endogenous E2 or repeated exposure to vaginal products containing E2 in vivo. The E2 concentrations range used in this study covers concentrations detected systemically in premenopausal women (up to ~ 500 pg/ml) as well as higher concentrations (up to 10,000 pg/ml), which are relevant considering intravaginally applied E2-containing products^20–23^.

The observed E2-mediated anti-HIV-1 activity in two model systems is consistent with previously reported (i) in vitro E2-mediated anti-HIV-1 activity in macrophages and CD4^+^ T cells (through induction of IFNα and β-catenin pathways)^19,24,25^; (ii) our ex vivo data in cervical explants showing direct association between serum E2 concentrations and cervical infection level^1^; (iii) data showing protective effect of vaginal E2-containing creams against cervical HIV-1 infection^26,27^; and (iv) in vivo data in non-human primates demonstrating protective effects of E2-containing implants and estriol cream against vaginal SIV challenge^28,29^. E2 was also reported to inhibit TCR activation of HIV-1 transcription through ESR1 and is likely to limit viral emergence from latency^30^. It needs to be noted that the stimulatory effect of E2 on HIV-1 transcription was also reported^31^.

In contrast to the initial hypothesis that was based on our RNAseq studies^2^, no consistent significant upregulation in CFL1 mRNA expression in PBMCs and endocervix was observed. This could have been due to selection of time points and/or effect of post-transcriptional regulation. We aimed to have parallel mRNA and protein expression data and selected time points starting from 48 h to days after E2 exposure. Analysis of earlier time points was not performed. As the analysis was done either in uninfected or HIV-1_BaL_ infected PBMCs, we cannot exclude effect of HIV-1_BaL_ infection on CFL1 mRNA expression.

It is well established that HIV-1 binding to blood CD4^+^ T cells leads to temporal changes in actin polymerization and depolymerization mediated by CFL1 inhibition through phosphorylation and activation through dephosphorylation, regulating HIV infection^4–6^. We speculate that the detected increase in total CFL1, pCFL1 and increased pCFL1/CFL ratio in PBMCs and endocervix following E2 exposure may impair the dynamic cytoskeletal treadmill by locking CFL1 in inactivated state in HIV target cells, and therefore, provide protection against infection. Future studies addressing E2-induced changes in CFL1 expression in specific HIV target cells are warranted.

Treatment of PBMCs and endocervical explants with LIMKi3 (specific inhibitor of CFL1 phosphorylation targeting LIMK1 and 2; no off-targets have been described^32^) reverted E2-mediated anti-HIV-1 activity. LIMKi3 reverted E2-induced increase in total CFL1 expression, pCFL1 expression and an increase in pCFL1/CFL1 ratios. These data are consistent with published data demonstrating involvement of LIMK in regulation of E2-mediated CFL1 phosphorylation in hippocampal neurons^33^. The data further implicate changes in CFL1 protein expression and pCFL1/CFL1 ratio in E2-mediated anti-HIV-1 activity. Lack of E2-mediated increase in total CFL1 expression in the presence of LIMKi3 requires further investigation.

CFL1 knockdown experiments in PBMCs demonstrated partial reversal of E2-mediated anti-HIV-1 activity. These data confirm involvement of CFL1 in E2-induced signaling leading to inhibition of HIV-1 infection. The partial effect is likely attributed to transient nature of CFL1 knockdown.

Our results support recent data implicating pCFL1 as potential biomarker of HIV-1 susceptibility and disease progression^6,13^. Intriguingly, blocking of HIV-induced LIMK1 activity was previously shown to inhibit HIV-1 entry, nuclear migration, viral release, and cell–cell transmission^7,34,35^. Our results contrast with these findings as LIMKi3 inhibited E2-mediated anti-HIV-1 activity and point to potentially different effects of stimuli (HIV-1 vs. E2) as well as LIMKs (LIMK1 vs. LIMK 1 and 2) on HIV-1 infection.

Analysis of CC/CK profile in supernatants from E2-treated HIV-1_BaL_ infected endocervical explants revealed an expected increase in concentrations of several mediators (IL1RA, IL-7, CXCL8, CXCL10, GM-CSF) over the course of the culture period. A decrease in CXCL10 and CCL5 after E2 exposure suggests an anti-inflammatory effect, which could have contributed to the observed anti-HIV-1 activity. CXCL10 was previously identified as a potent marker of high HIV-1 acquisition risk as high genital concentrations in seronegative women were found to be associated with higher subsequent infection^36–38^. CCL5 is a proinflammatory chemokine which attracts HIV-1 target cells to the mucosal sites and is increased in vaginal secretions of women at the higher risk of infection^39–41^. High CCL5 in vaginal fluids was shown to correlate with high HIV-1 seroconversion rate^39^.

Our study has several limitations. We did not explore effects of different E2 exposure regimens on HIV-1_BaL_ infection and CFL1 expression as we focused on one regimen mimicking continuous exposure to E2. We also did not explore if progesterone might impact observed E2-mediated activity. Given the dynamic changes in progesterone and E2 concentrations during menstrual cycle, potential impact of progesterone on E2-induced changes in CFL1 expression and anti-HIV-1 activity deserves further exploration.

Overall, our results suggest a link between E2-mediated anti-HIV-1 activity, increased CFL1 expression and inactivation through phosphorylation in PBMCs and endocervical mucosa. It needs to be acknowledged that estrogen-only therapy is associated with a number of risks for women, and, therefore, use of unopposed E2 for HIV-1 prevention is problematic^42^. However, our data support exploration of targeted manipulation of CFL1 for HIV-1 prevention.

## Supplementary Information

Supplementary Information.

## Data Availability

All relevant data are within the Manuscript and its Supporting Information files.
