# Supplementary material for: Estradiol inhibits HIV-1BaL infection and induces CFL1 expression in peripheral blood mononuclear cells and endocervical mucosa
Source: Sci Rep. 2022 Apr 13;12:6165. doi: 10.1038/s41598-022-10163-6 (PMC9008051; doi:10.1038/s41598-022-10163-6)
Supplement: Supplementary file 1 — Supplementary Information. [file 41598_2022_10163_MOESM1_ESM.pdf]

Estradiol inhibits HIV-1$_{BaL}$ infection and induces CFL1 expression in peripheral blood mononuclear cells and endocervical mucosa

N. Verma, S. Mukhopadhyay, P. Barnable, M. G. Plagianos, N. Teleshova*

Affiliation:

Center for Biomedical Research, Population Council, 1230 York Ave. New York, NY, 10065, USA.

*To whom correspondence should be addressed: Natalia Teleshova, nteleshova@popcouncil.org

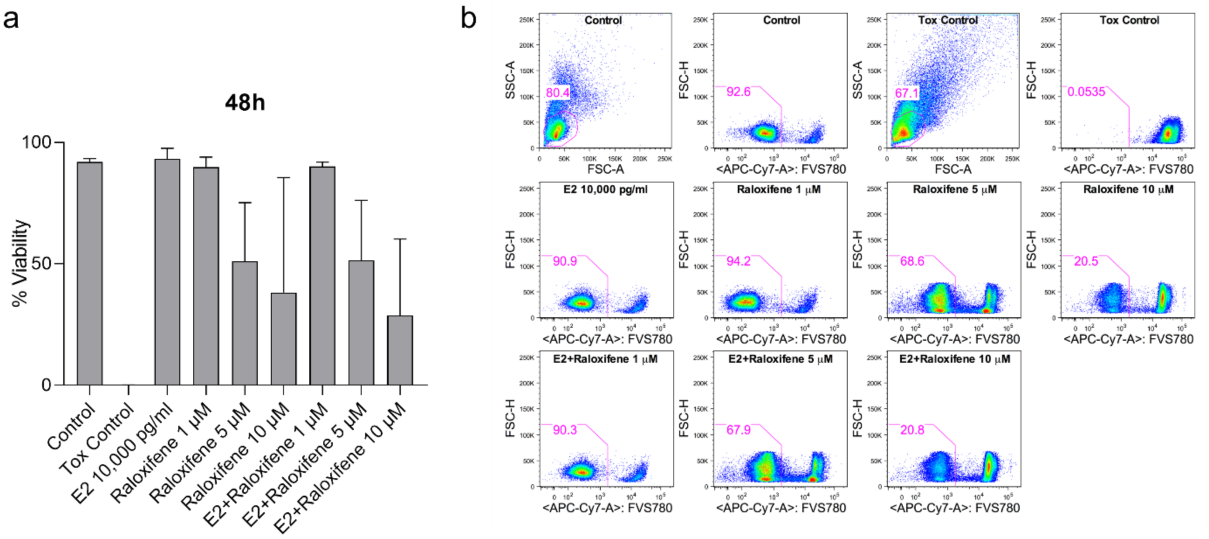

Figure S1. Effect of E2 and Raloxifene on PBMCs viability. PBMCs were incubated with Raloxifene for 3 h followed by 48 h incubation with E2 10,000 pg/ml (E2). Untreated PBMCs (Control) and 16% PFA treated PBMCs (Toxicity Control) were included. PBMCs were stained with FVS780, acquired on BD LSR II and analyzed by FLOWJO 8.8.6 software. Shown is **(a)** the summary of 4 experiments (Mean±SEM) and **(b)** representative dot plots from a single experiment.

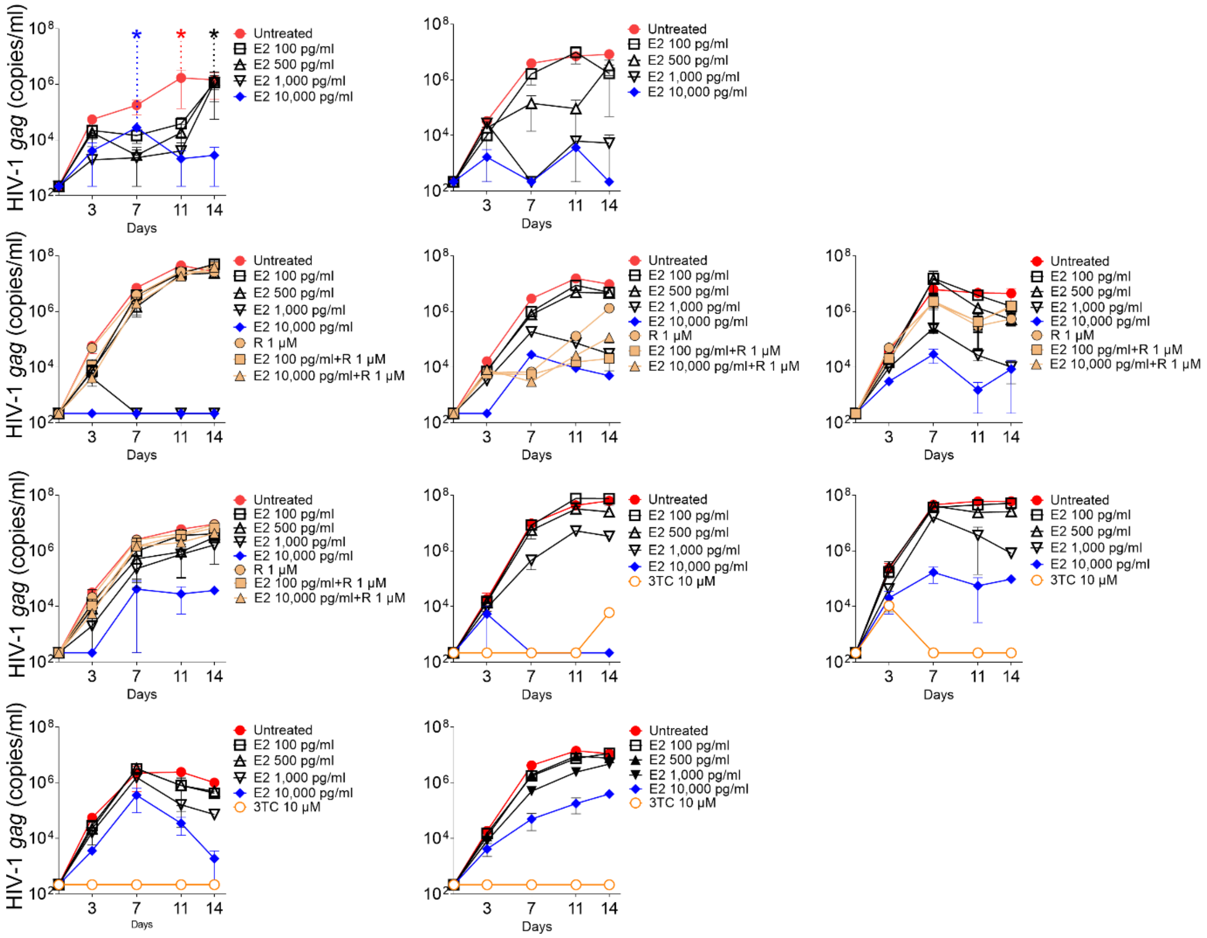

Figure S2a. E2 inhibits HIV-1$_{BaL}$ infection in PBMCs. PBMCs were incubated with E2 (vs. untreated control) for 48 h followed by 1,000 $TCID_{50}/10^6$ cells HIV-1$_{BaL}$ challenge and then cultured for 14 days with repeated addition of E2 on days 3, 7 and 11. Raloxifene (R) and 3TC were included in selected experiments. Shown are viral growth kinetics (HIV-1 *gag* copy numbers/ml) over 14 days of culture in individual experiments (Mean±SEM of replicates). SOFT endpoints in a single experiment are shown for illustrative purposes. *=SOFT untreated; *=SOFT E2 10,000 pg/ml; *= SOFT E2 100, 500 and 1,000 pg/ml.

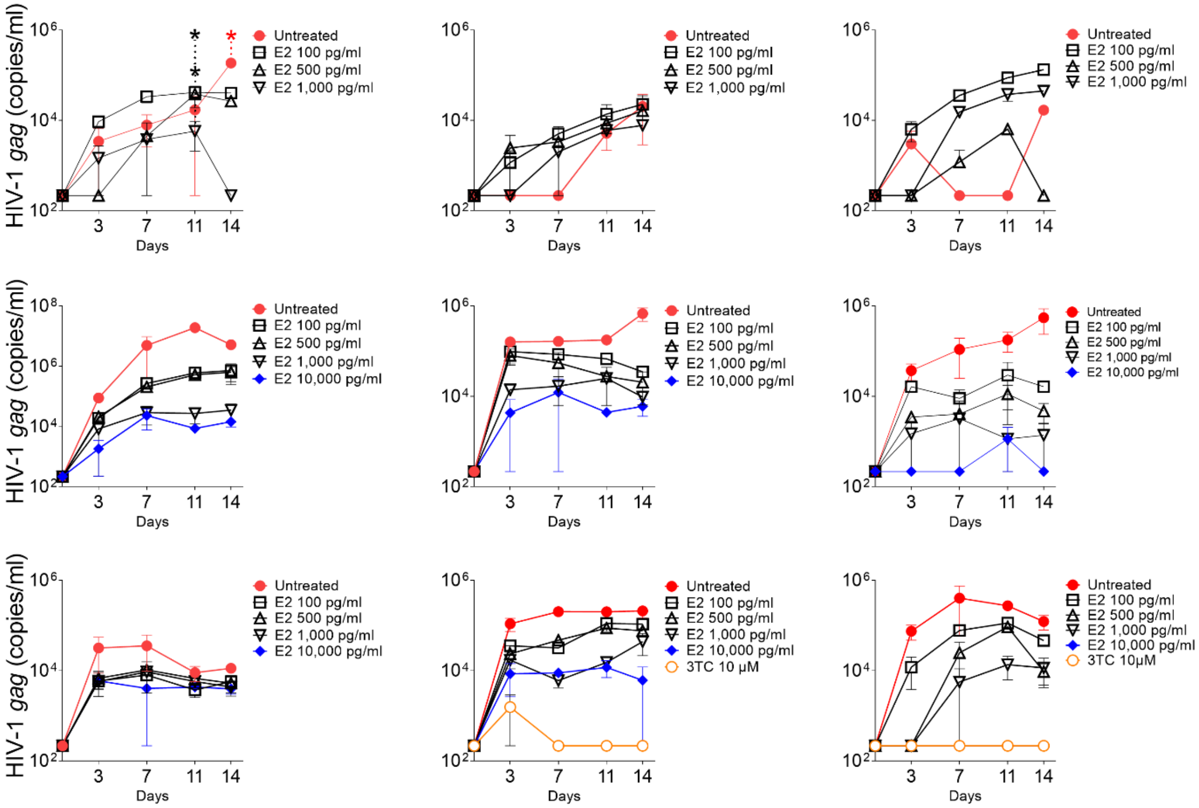

Figure S2b. E2 inhibits HIV-1$_{BaL}$ infection in endocervix. Endocervical explants were incubated with E2 (vs. untreated control) for 48 h followed by 500 $TCID_{50}$/explant HIV-1$_{BaL}$ challenge and then cultured for 14 days with repeated addition of E2 on days 3, 7 and 11. 3TC was included in selected experiments. Shown are viral growth kinetics (HIV-1 *gag* copy numbers/ml) over 14 days of culture in individual experiments (Mean±SEM of replicates). SOFT endpoints in a single experiment are shown for illustrative purposes. *=SOFT untreated; *=SOFT E2 100, 500 and 1,000 pg/ml.

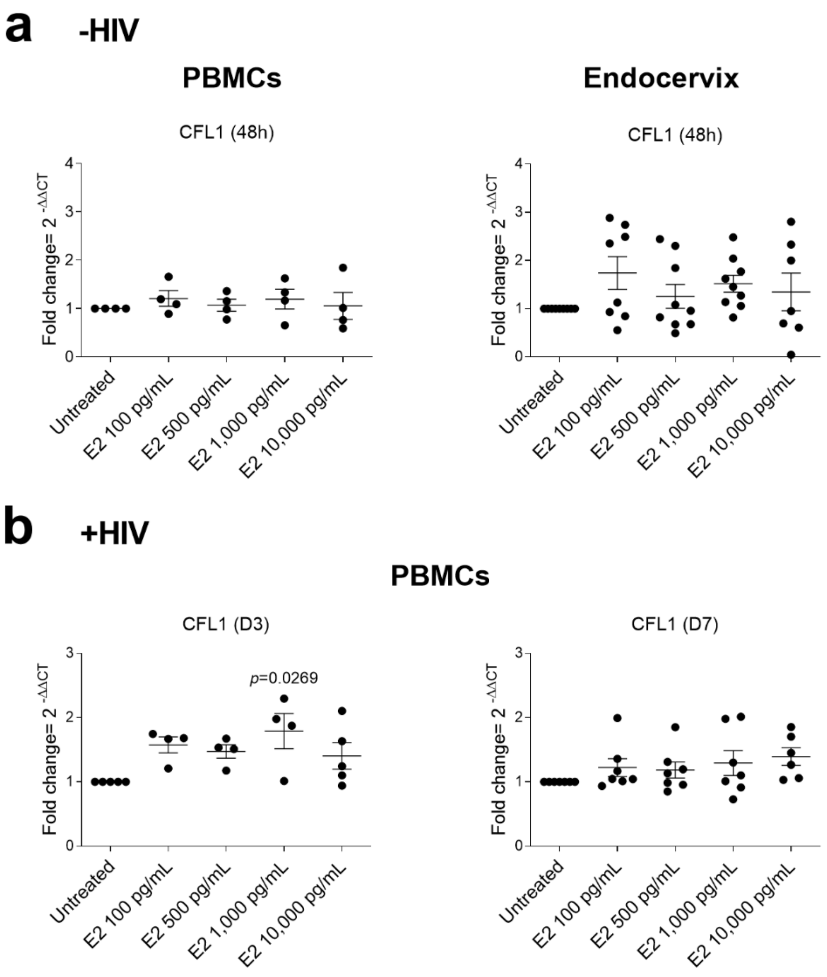

Figure S3. Effect of E2 on CFL1 mRNA expression in PBMCs and endocervix. (a) PBMCs and endocervical explants were incubated with E2 (vs. untreated control) for 48 h or **(b)** incubated with E2 (vs. untreated control), challenged with 1,000 $TCID_{50}/10^6$ cells HIV-1_{BaL} and cultured for 3 or 7 days. CFL1 mRNA expression was measured by qPCR. Shown are the changes in mRNA expression relative to untreated controls (Mean±SEM; each symbol represents individual experiment).

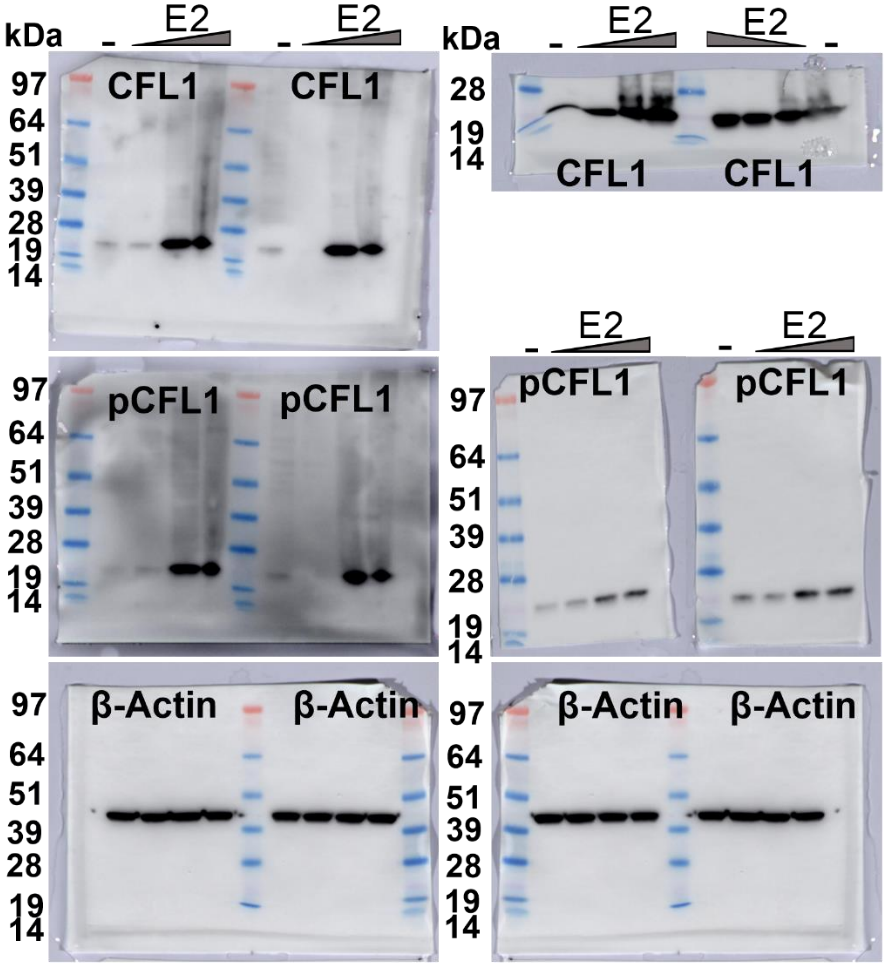

Figure S4a. E2 induces CFL1 expression in PBMCs. Shown are uncropped blots of individual experiments presented in Fig. 6a. PBMCs were incubated with E2 100, 1,000, 10,000 pg/ml (vs. untreated control) for 48 h (4 experiments). WCEs (60 µg) were probed for CFL1 and pCFL1. β-actin (60 µg) was used as internal control.

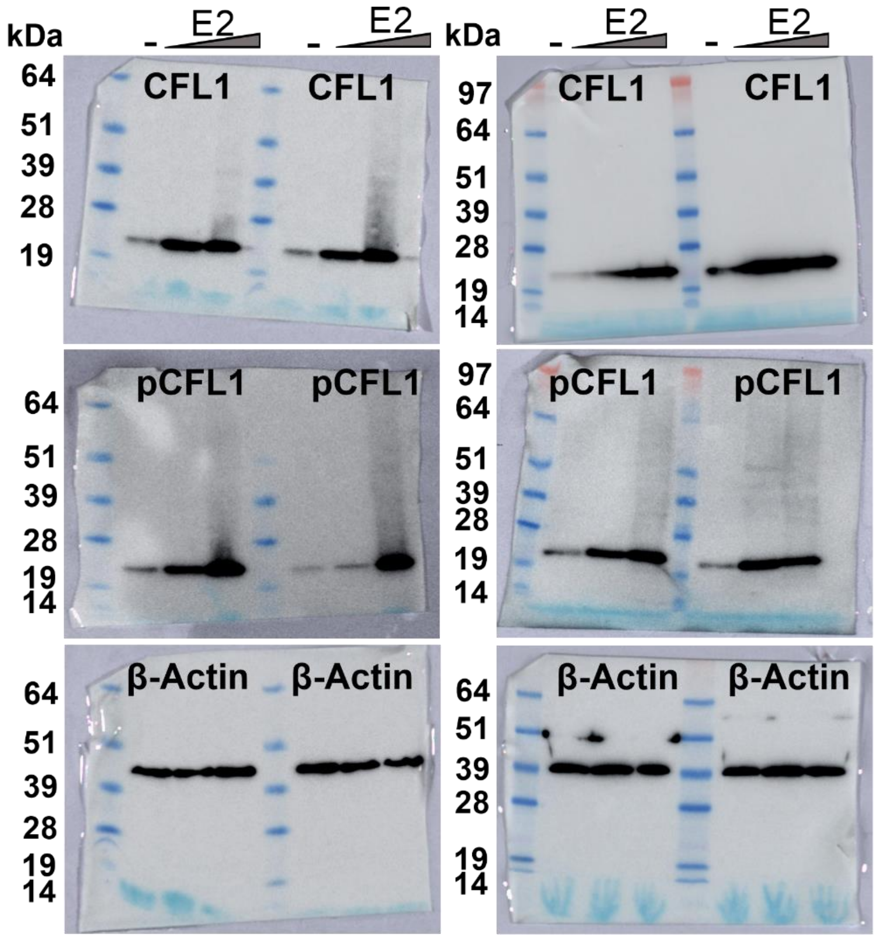

Figure S4b. E2 induces CFL1 expression in PBMCs. Shown are uncropped blots of individual experiments presented in Fig. 6b. PBMCs were incubated with E2 100 or 10,000 pg/ml for 48 h (vs. untreated control), challenged with 1,000 $TCID_{50}/10^6$ cells HIV-1_{BaL} and cultured for 7 days (4 experiments). WCEs (60 µg) were probed for CFL1 and pCFL1. β-actin (60 µg) was used as internal control.

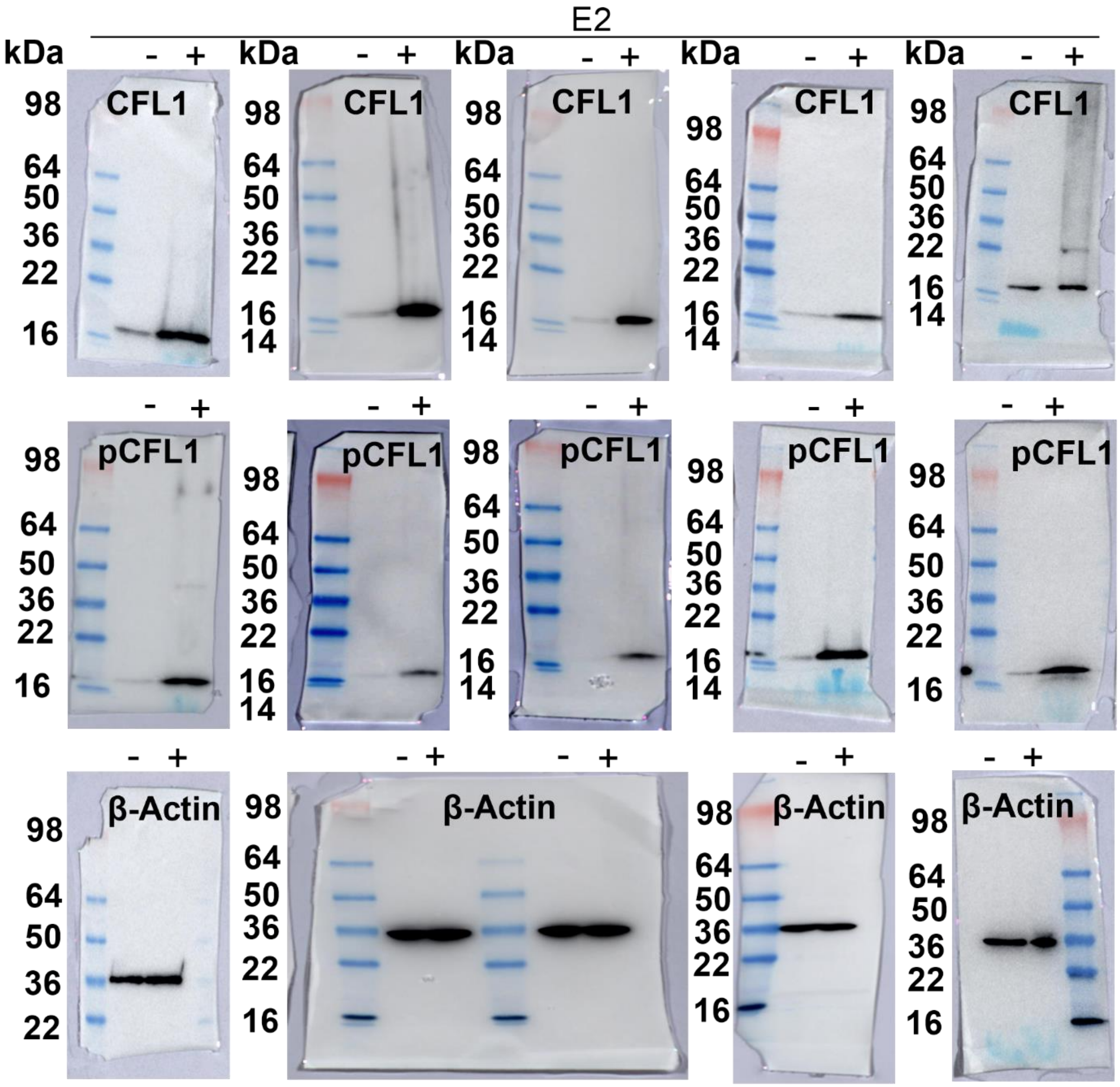

Figure S4c. E2 induces CFL1 expression in endocervix. Shown are uncropped blots of individual experiments presented in Fig. 6c. Endocervical explants were incubated with E2 10,000 pg/ml (vs. untreated control) for 48 h (5 experiments). WCEs (60-120 µg) were probed for CFL1 and pCFL1. β-actin (60-120 µg) was used as internal control.

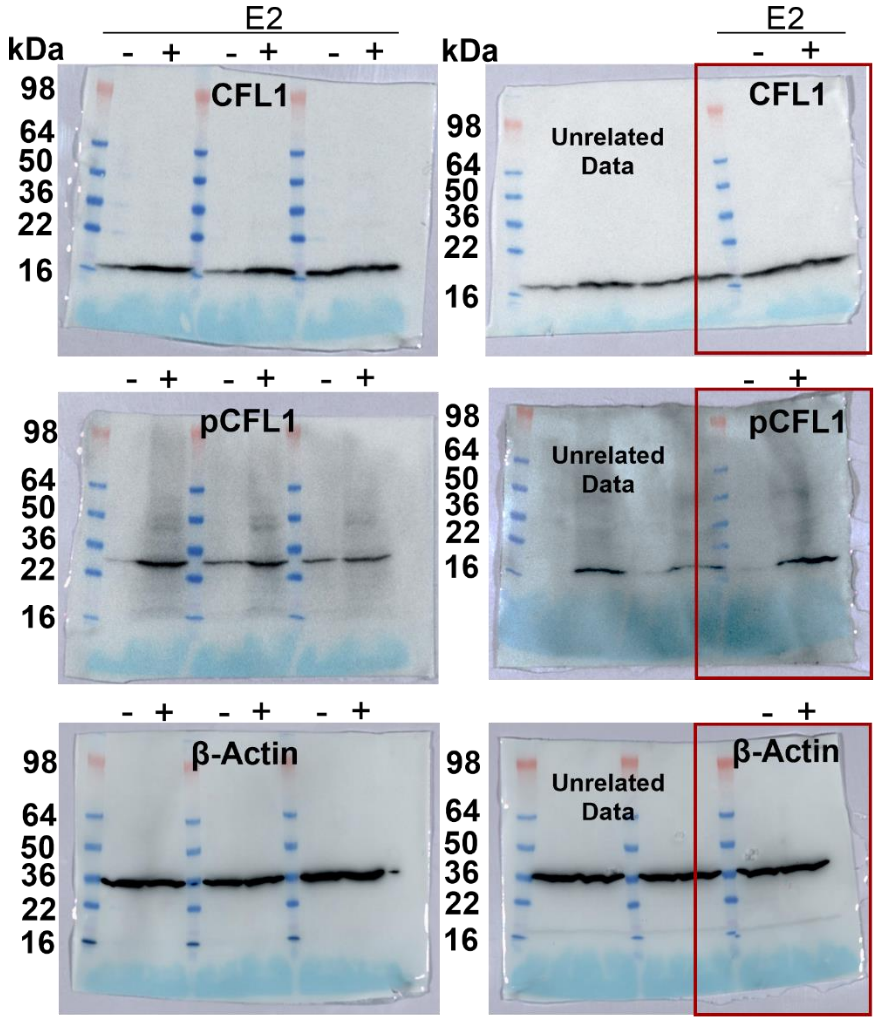

Figure S4d. E2 induces CFL1 expression in PBMCs and endocervix. Shown are uncropped blots of individual experiments presented in Fig. 6d. Endocervical explants were incubated with E2 10,000 pg/ml (vs. untreated control) for 48 h, challenged with 500 $TCID_{50}$ HIV-1_{BaL} and cultured for 7 days (4 experiments). WCEs (60-120 µg) were probed for CFL1 and pCFL1. β-actin (60-120 µg) was used as internal control.

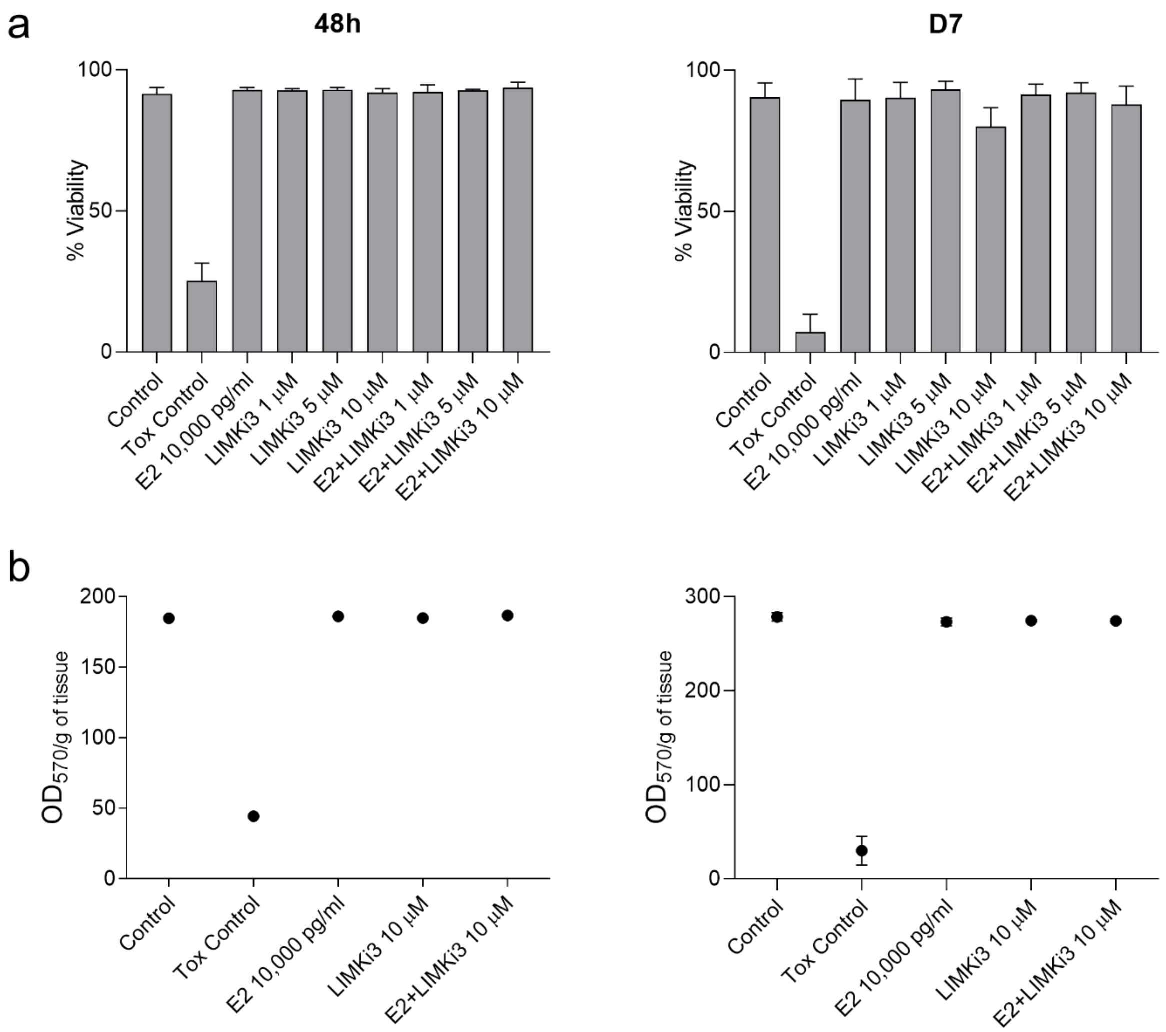

Figure S5. Effect of LIMKi3 on PBMCs and endocervix viability. (a) PBMCs were incubated with LIMKi3 for 3 h prior to 48 h incubation with E2 10,000 pg/ml (E2) followed by 1,000 $TCID_{50}/10^6$ cells HIV-1_{BaL} challenge and 7 days culture. Untreated PBMCs (Control) and 16% PFA (Toxicity Control) conditions were included. PBMCs were collected before challenge with HIV-1_{BaL} (48 h time point) and on day 7 of culture (D7) post HIV-1_{BaL} challenge, stained with FVS780, acquired on BD LSR II instrument, and analyzed by FLOWJO 8.8.6 software. The summaries of 3-4 experiments (Mean±SEM) are shown. **(b)** Endocervical explants were incubated with LIMKi3 for 3 h prior to 48 h incubation with E2. Untreated explants (Control) and 16% PFA treated explants (Toxicity Control) conditions were included. Tissue viability was determined by MTT assay. Shown are two experiments (Mean±SEM of OD_{570}/g of tissue for each condition (duplicates).

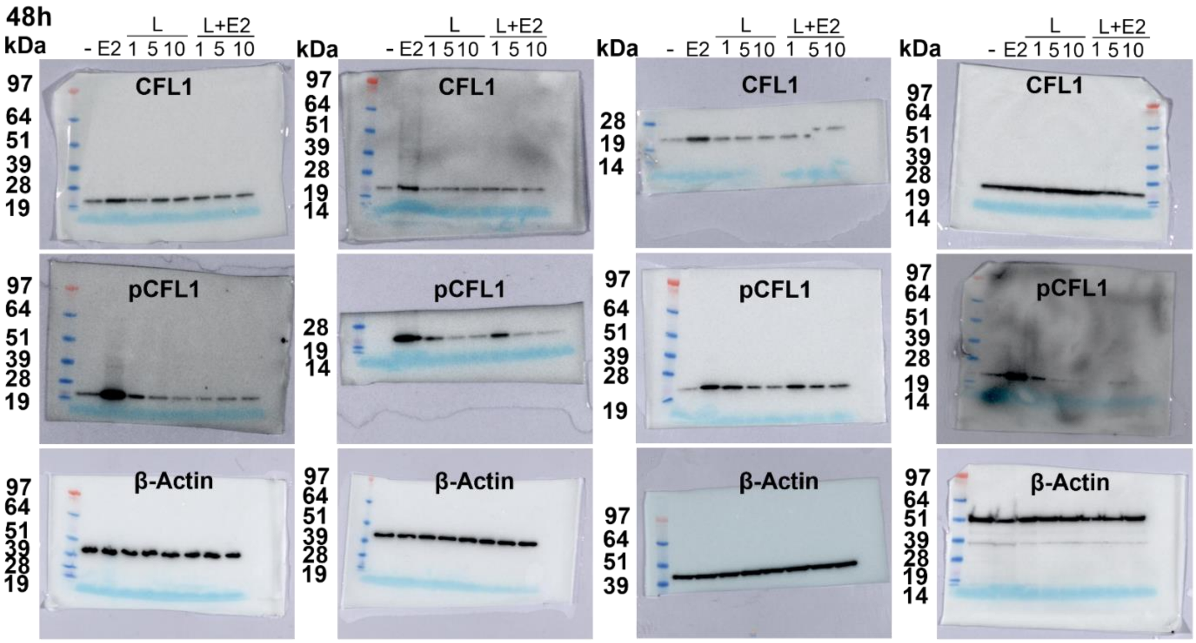

Figure S6a. Effect of LIMKi3 on CFL1 and pCFL1 expression in PBMCs. Shown are uncropped blots of individual experiments presented in Fig. 7b 48 h panel. PBMCs were incubated with 1-10 μM LIMKi3 (L) for 3 h followed by incubation with or without E2 10,000 pg/ml (E2) for 48h. Untreated and E2 treated conditions were included. WCEs (60 μg) were prepared and probed for CFL1 and pCFL1. β-actin (60 μg) was used as internal control.

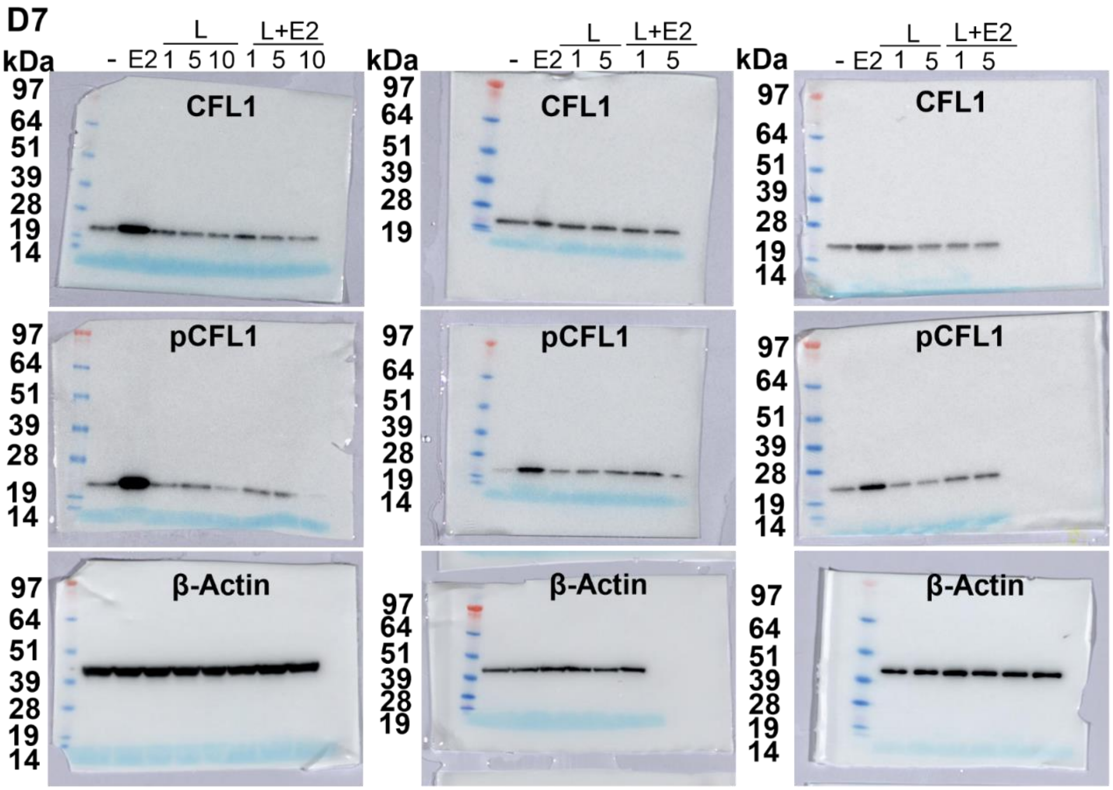

Figure S6b. Effect of LIMKi3 on CFL1 and pCFL1 expression in PBMCs. Shown are uncropped blots of individual experiments presented in Fig. 7b D7 panel. PBMCs were incubated with 1-10 µM LIMKi3 (L) with or without E2 10,000 pg/ml (E2) for 48 h followed by HIV-1_{BaL} challenge and culture for 7 days. Untreated and E2 treated conditions were included. WCEs (60 µg) were prepared and probed for CFL1 and pCFL1. β-actin (60 µg) was used as internal control.

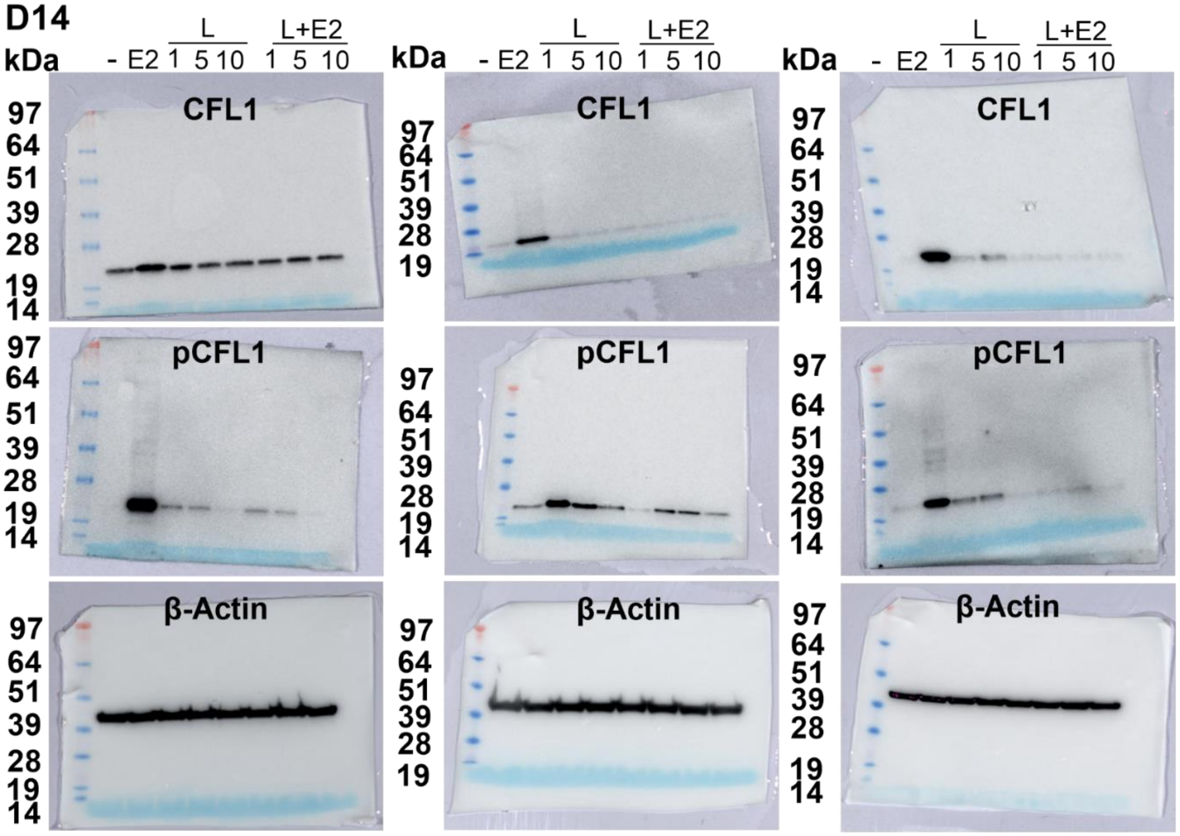

Figure S6c. Effect of LIMKi3 on CFL1 and pCFL1 expression in PBMCs. Shown are uncropped blots of individual experiments presented in Fig. 7b D14 panel. PBMCs were incubated with 1-10 μM LIMKi3 (L) with or without E2 10,000 pg/ml (E2) for 48 h followed by HIV-1_{BaL} challenge and 14 days culture. Untreated and E2 treated conditions were included. WCEs (60 μg) were prepared and probed for CFL1 and pCFL1. β-actin (60 μg) was used as internal control.

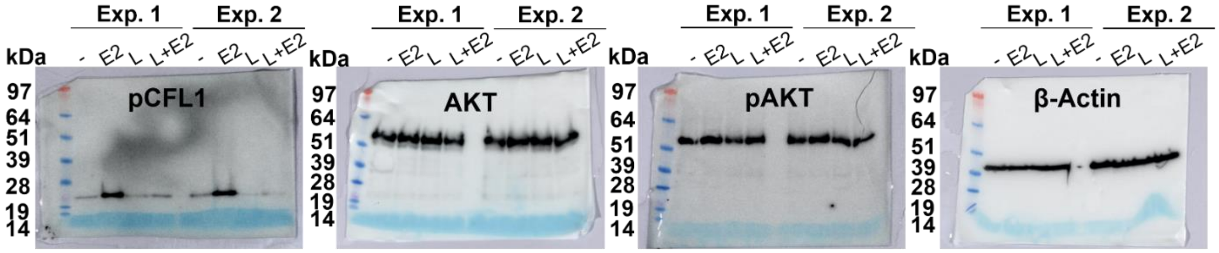

Figure S7. Specificity of LIMKi3 in PBMCs. PBMCs were treated with E2 10,000 pg/ml (E2), LIMKi3 10 μM (L) or L+E2 (vs. untreated control) for 48 h. WCEs were prepared and probed for pCFL1 (60 μg), panAKT (60 μg) and pAKT (120 μg) Abs to confirm specificity of LIMKi3. Shown are uncropped original blots of two individual experiments.

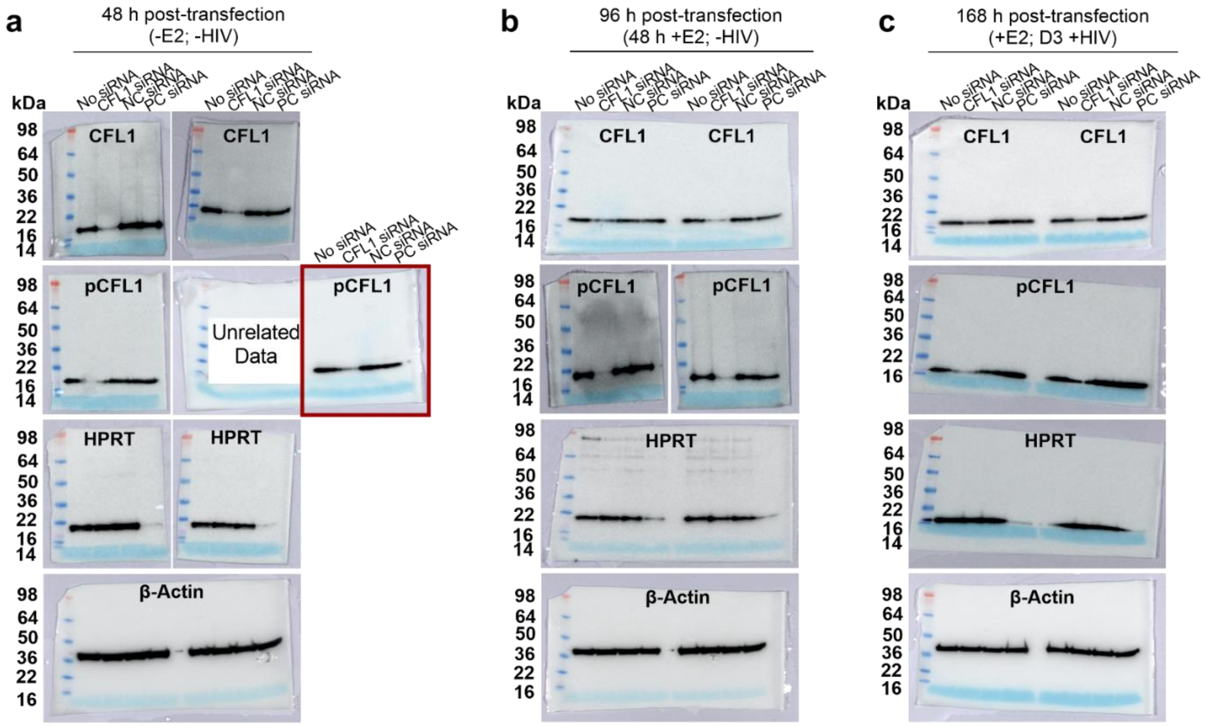

Figure S8. Knockdown of CFL1 reduces total CFL1 and pCFL1 protein expression in PBMCs. Shown are uncropped blots of two individual experiments presented in Fig. 8a**.** PBMCs were transfected with CFL1 SiRNA, Trilencer-27 Universal scrambled negative control siRNA duplex and Trilencer-27 HPRT Positive control siRNA duplex using Viromer Green transfection reagent. 48 h post-transfection the cells were incubated with E2 10,000 pg/ml (E2) for 48 h, challenged with 1,000 $TCID_{50}/10^6$ cells HIV-1_{BaL} and cultured for 3 days. WCEs (120 µg) were prepared at 48 h, 96 h and 168 h post-transfection and probed for CFL1 and pCFL1. β-actin (120 µg) was used as internal control. NC siRNA= Negative Control/Trilencer-27 Universal scrambled negative control siRNA duplex; PC siRNA= Positive Control/ Trilencer-27 HPRT Positive control siRNA duplex.

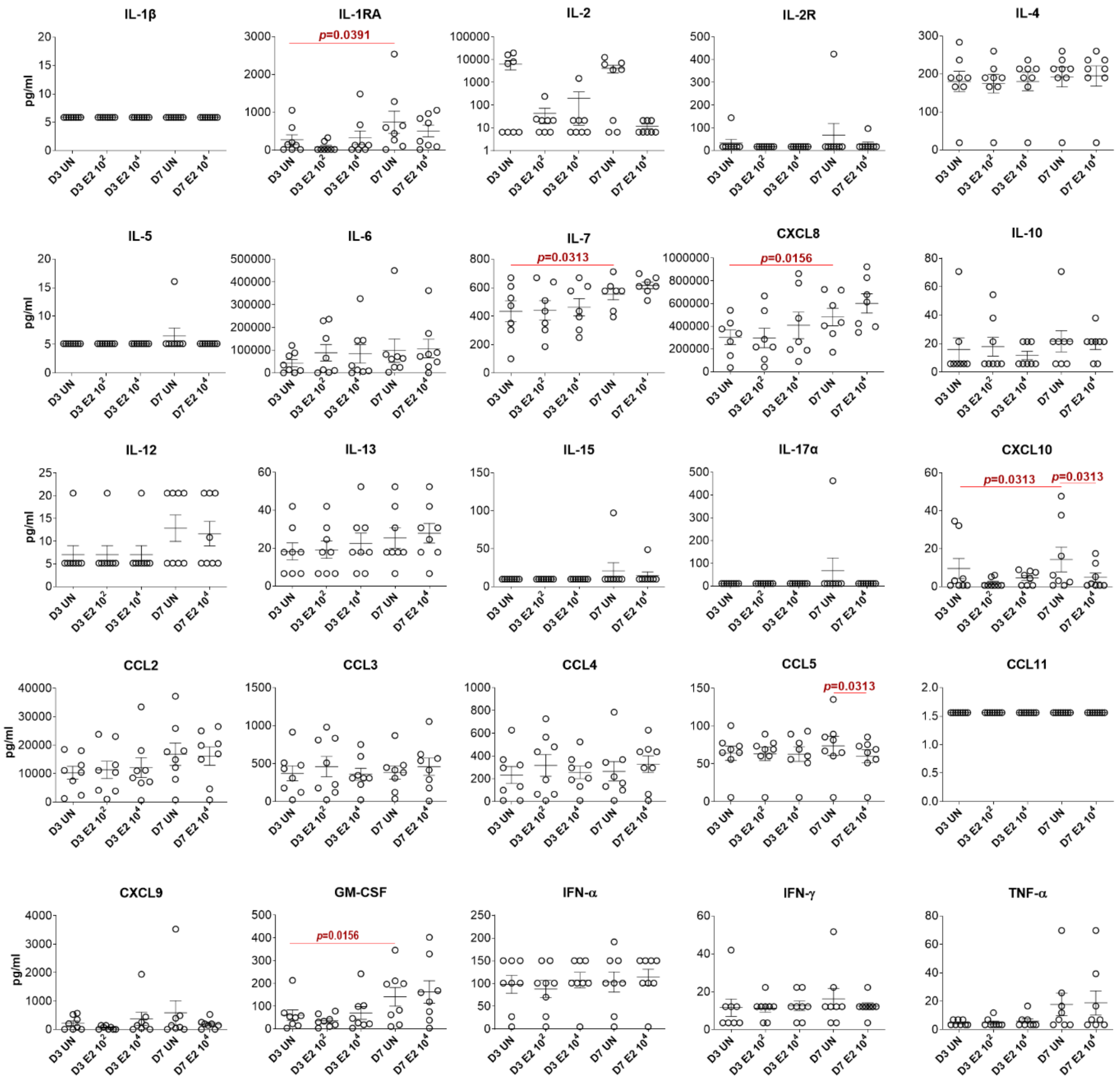

Figure S9. Effect of E2 treatment on CC/CK concentrations in endocervical explant cultures. CC/CK concentrations were measured in day 3 (D3) and day 7 (D7) tissue culture supernatants from experiments included in Fig. 1b/S2b using 25-plex Luminex kit. Shown are data from 8 experiments (Mean±SEM). Untreated (UN) condition, E2 100 pg/ml (E2 10^2) and E2 10,000 pg/ml (E2 10^4) conditions are included. Values <LLOQ were assumed LLOQ values.
